# GWAS of the electrocardiographic QT interval in Hispanics/Latinos generalizes previously identified loci and identifies population-specific signals

**DOI:** 10.1038/s41598-017-17136-0

**Published:** 2017-12-06

**Authors:** Raúl Méndez-Giráldez, Stephanie M. Gogarten, Jennifer E. Below, Jie Yao, Amanda A. Seyerle, Heather M. Highland, Charles Kooperberg, Elsayed Z. Soliman, Jerome I. Rotter, Kathleen F. Kerr, Kelli K. Ryckman, Kent D. Taylor, Lauren E. Petty, Sanjiv J. Shah, Matthew P. Conomos, Nona Sotoodehnia, Susan Cheng, Susan R. Heckbert, Tamar Sofer, Xiuqing Guo, Eric A. Whitsel, Henry J. Lin, Craig L. Hanis, Cathy C. Laurie, Christy L. Avery

**Affiliations:** 1 Department of Epidemiology, University of North Carolina, Chapel Hill, NC USA; 2 Department of Biostatistics, University of Washington, Seattle, WA USA; 3 The Vanderbilt Genetics Institute, Vanderbilt University Medical Center, Nashville, TN USA; 4 The Institute for Translational Genomics and Population Sciences and Department of Pediatrics, Los Angeles Biomedical Research Institute at Harbor-UCLA Medical Center, Torrance, CA USA; 5 Division of Epidemiology and Community, University of Minnesota, Minneapolis, MN USA; 6 Division of Public Health Sciences, Fred Hutchinson Cancer Research Center, Seattle, WA USA; 7 Department of Internal Medicine, Section on Cardiology, Wake Forest School of Medicine, Winston-Salem, NC USA; 8 Epidemiological Cardio Research Center (EPICARE), Department of Epidemiology and Prevention, Wake Forest School of Medicine, Winston-Salem, NC USA; 9 Departments of Epidemiology and Pediatrics, University of Iowa, Iowa City, IA USA; 10 Human Genetics Center, University of Texas, Health Science Center at Houston, Houston, TX USA; 11 Center for Precision Medicine, University of Texas, Health Science Center at Houston, Houston, TX USA; 12 Division of Cardiology, Bluhm Cardiovascular Institute, Northwestern Memorial Hospital, Northwestern University Feinberg School of Medicine, Chicago, IL USA; 13 Cardiovascular Health Research Unit, University of Washington, Seattle, WA USA; 14 Division of Cardiology, Department of Medicine, University of Washington, Seattle, WA USA; 15 Brigham and Women’s Hospital, Division of Cardiovascular Medicine, Boston, MA USA; 16 Department of Epidemiology, University of Washington, Seattle, WA USA; 17 Department of Medicine, Harvard Medical School, Boston, MA USA; 18 Division of Sleep and Circadian Disorders, Brigham and Women’s Hospital, Boston, MA USA; 19 Department of Medicine, University of North Carolina, Chapel Hill, NC USA; 20 Division of Medical Genetics, Harbor-UCLA Medical Center, Torrance, CA USA; 21 Carolina Population Center, University of North Carolina, Chapel Hill, NC USA

## Abstract

QT interval prolongation is a heritable risk factor for ventricular arrhythmias and can predispose to sudden death. Most genome-wide association studies (GWAS) of QT were performed in European ancestral populations, leaving other groups uncharacterized. Herein we present the first QT GWAS of Hispanic/Latinos using data on 15,997 participants from four studies. Study-specific summary results of the association between 1000 Genomes Project (1000G) imputed SNPs and electrocardiographically measured QT were combined using fixed-effects meta-analysis. We identified 41 genome-wide significant SNPs that mapped to 13 previously identified QT loci. Conditional analyses distinguished six secondary signals at *NOS1AP* (n = 2), *ATP1B*1 (n = 2), *SCN5A* (n = 1), and *KCNQ1* (n = 1). Comparison of linkage disequilibrium patterns between the 13 lead SNPs and six secondary signals with previously reported index SNPs in 1000G super populations suggested that the *SCN5A* and *KCNE1* lead SNPs were potentially novel and population-specific. Finally, of the 42 suggestively associated loci, *AJAP1* was suggestively associated with QT in a prior East Asian GWAS; in contrast *BVES* and *CAP2* murine knockouts caused cardiac conduction defects. Our results indicate that whereas the same loci influence QT across populations, population-specific variation exists, motivating future trans-ethnic and ancestrally diverse QT GWAS.

## Introduction

The QT interval (QT), as measured by the resting 12-lead electrocardiogram (ECG), provides a non-invasive assessment of ventricular repolarization, the prolongation or shortening of which is an established risk factor for a spectrum of cardiovascular diseases, including sudden cardiac death (SCD)^1^. Although SCD accounts for roughly 10–20% of total mortality in industrial countries, prevention and treatment remains incomplete, resulting in a majority of cases occurring in the absence of clinical features that would elicit medical attention^2^. Additional efforts to understand underlying biology are therefore needed.

QT genome-wide association studies (GWAS) provide a means at informing SCD biology, if not prevention and treatment^3^, because approximately 10% SCD cases are caused by *torsades de pointes* ^4^. QT also is heritable^5^ and reliably measured^6^. Moreover, GWAS-identified QT SNPs have been associated with a >30% increase in risk of SCD, results replicated by some studies^7,8^, but not others^9,10^. In contrast, SCD GWAS have been difficult to perform, likely reflecting the small sample sizes, phenotypic heterogeneity, and outcome measurement error that characterize existing studies^11^, therefore resulting in a limited number of loci identified to-date^7,12^. Together, these findings motivate additional, well-powered GWAS of QT to improve understanding of QT prolongation and SCD.

Currently a majority of GWAS of QT have been conducted in populations of European ancestry^13–19^, although modestly sized studies of African Americans^20,21^ and East Asians^22,23^ also have been published. Few QT GWAS have included Hispanic/Latino populations, which will constitute 31% of the U.S. population by 2060^24^ and shoulder increased burdens of QT prolonging and SCD-predisposing obesity and diabetes as compared to European ancestral populations^25,26^. Here we present the first QT GWAS of Hispanic/Latinos.

## Results

This GWAS included 15,997 individuals of Hispanic/Latino ancestry from four cohorts ranging in size from 883 to 11,932 participants. Study participants were predominantly female (64%), middle aged (mean = 49 years), and obese (mean body mass index = 30 kg/m^2^) (Supplementary Table 1). The prevalence of diabetes ranged from 8.0% (Women’s Health Initiative, WHI) to 45.6% (Starr County, reflecting a study design with approximately equal proportions of participants with and without diabetes).

### Genome-wide Association Analysis

**Figure 1:**
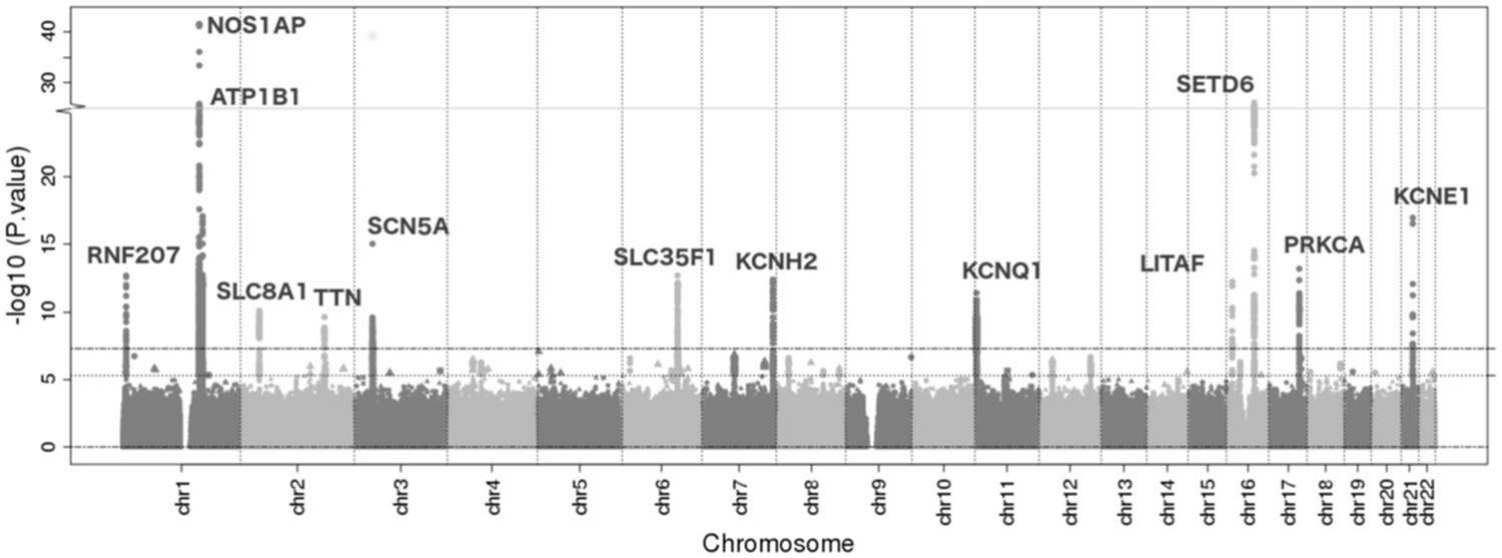
Manhattan plot of results from QT GWAS of 15,997 participants of Hispanic/Latino ancestry. The y-axis represents −log10(P-value) and truncated at 25. SNPs, ordered by chromosome and position, are shown on the x-axis. Significant loci are labelled as the nearest gene.

After study-specific quality control and filtering by effective sample size (see Methods), studies contributed between 5,997,534 (Starr County) and 17,322,742 (Hispanic Community Health Study/Study of Latinos, HCHS/SOL) imputed SNPs (Supplementary Table 2), which together represented 17,586,686 unique SNPs. A total of 41 SNPs at 13 of the 35 previously identified QT loci^19^ were genome wide significant (Fig. 1, Table 1 and Supplementary Table 3), with no evidence of genomic inflation (study-specific λ range: 0.98–1.02, Supplementary Figures 1 and 2; λ = 1.01). A total of 42 suggestive loci (P-val < 5 × 10^−6^) were also identified (Supplementary Table 4); notably, 26 of the 42 suggestive loci only passed the effective sample size filter in the HCHS/SOL study, likely reflecting their rarity (i.e. minor allele frequency [MAF] < 0.05). Both genome-wide significant and suggestive loci demonstrated wide variation in minor allele frequency across ancestries (Supplementary Tables 3, 4), although very limited reporting of suggestive loci or publication of GWAS summary statistics from imputed data limited comprehensive evaluation of suggestive loci.

**Table 1:** Genome-wide significant loci identified in a GWAS meta-analysis of n = 15,997 participants of Hispanic/Latino ancestry from four studies, that were previously reported.

Locus	Lead SNP	Chr	Position (hg19)	A1	A2	CAF	β (ms)	Direction of β	SE (ms)	P-val	P_het_
*RNF207*	rs7531322	1	6,299,823	C	G	0.30	1.73	++++	0.24	1.04e–12	0.17
*NOS1AP*	rs12143842	1	162,033,890	T	C	0.22	3.46	++++	0.25	3.30e–42	0.35
*ATP1B1*	rs12035622	1	169,102,340	A	T	0.19	−2.36	−−−−	0.27	8.77e–18	0.73
*SLC8A1*	rs35450971	2	40,754,314	T	C	0.79	1.71	++++	0.26	8.37e–11	0.45
*TTN*	rs55863869	2	179,647,546	A	G	0.84	−1.92	−−−−	0.30	2.40e–10	0.42
*SCN5A*	rs3922844	3	38,624,253	T	C	0.37	1.77	++++	0.22	9.52e–16	0.06
*SLC35F1*	rs2078383	6	118,706,643	T	C	0.25	1.83	++++	0.25	2.07e–13	0.82
*KCNH2*	rs35760656	7	150,658,678	A	G	0.35	1.70	++++	0.23	4.21e–13	0.50
*KCNQ1*	rs12271931	11	2,478,519	A	G	0.93	3.92	??++	0.57	4.07e–12	0.13
*LITAF*	rs735951	16	11,693,536	A	G	0.41	−1.55	−−−−	0.22	5.92e–13	0.78
*SETD6*	rs185639574	16	58,550,052	T	G	0.34	−2.53	−−−−	0.24	6.67e–27	0.52
*PRKCA*	rs56152251	17	64,280,153	A	G	0.44	−1.60	−−−−	0.21	6.64e–14	0.85
*KCNE1*	rs12626657	21	35,828,173	A	G	0.15	2.69	+++−	0.31	1.14e–17	0.01

Chr: chromosome number. Position: base pair position in Build 37 (hg19). A1, A2: coded/non-coded alleles. β: effect estimate in ms. Direction of β: direction of the effect estimates per study following this order: WHI, MESA, HCHS/SOL and Starr County; ‘?’ means the SNP is not present in that particular study. SE: standard error. P_het_: P-val for Cochran’s Q test of homogeneity among cohorts.

For the 13 lead (i.e. locus-specific and most significant) SNPs in previously detected QT loci, little evidence of heterogeneity among studies was detected (Cochran’s Q test P-val > 0.05) and study-specific estimates exhibited directional consistency in estimated effects with the exception of rs12626657 at the *KCNE1* locus. Eleven of the 13 lead SNPs were correlated (r^2^ > 0.20; Supplemental Table 5; LD calculated separately in 1000G African [AFR], Ad Mixed American [AMR], East Asian [ASN], and European [EUR] super populations) with previously reported genome-wide significant index SNPs. However, the *SCN5A* (rs3922844) and *KCNE1* (rs12626657) Hispanic/Latino lead SNPs demonstrated little correlation with previously reported QT lead SNPs. *KCNE1* lead SNP rs12626657 (Hispanic/Latino MAF = 0.15, Table 1) also was monomorphic in the EUR 1000 Genomes super population.

**Figure 2:**
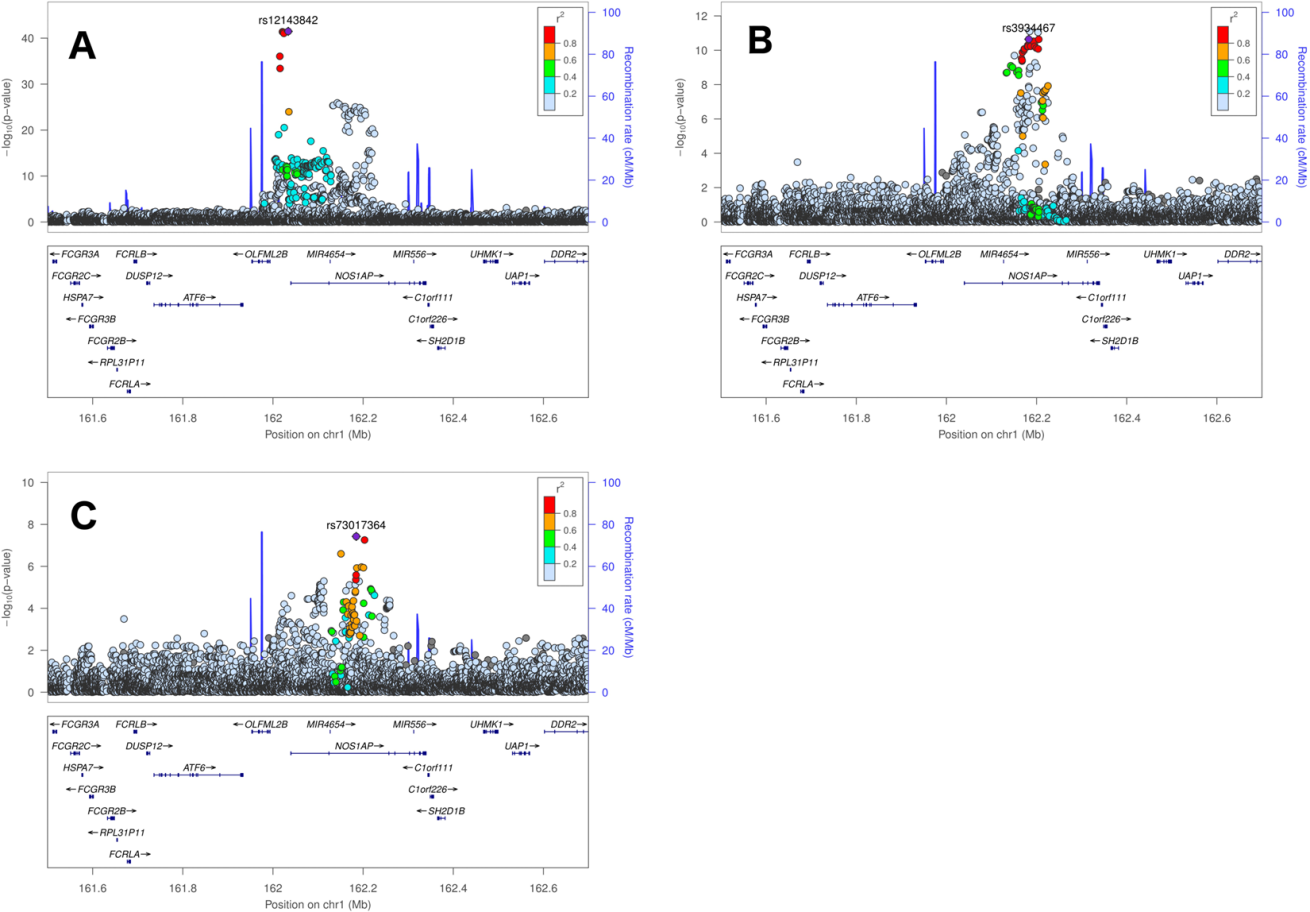
Locus zoom plots of the *NOS1AP* gene region showing SNP p-values from the primary (**A**) and conditional analyses (**B**,**C**). The lead SNP in the primary analysis is the previously reported rs12143842 (panel A), the secondary lead SNP after conditioning on rs12143842 is rs3934467 (panel B), and the secondary lead SNP after conditioning on rs12143842 and rs3934467 simultaneously is rs73017364 (panel C).

**Figure 3:**
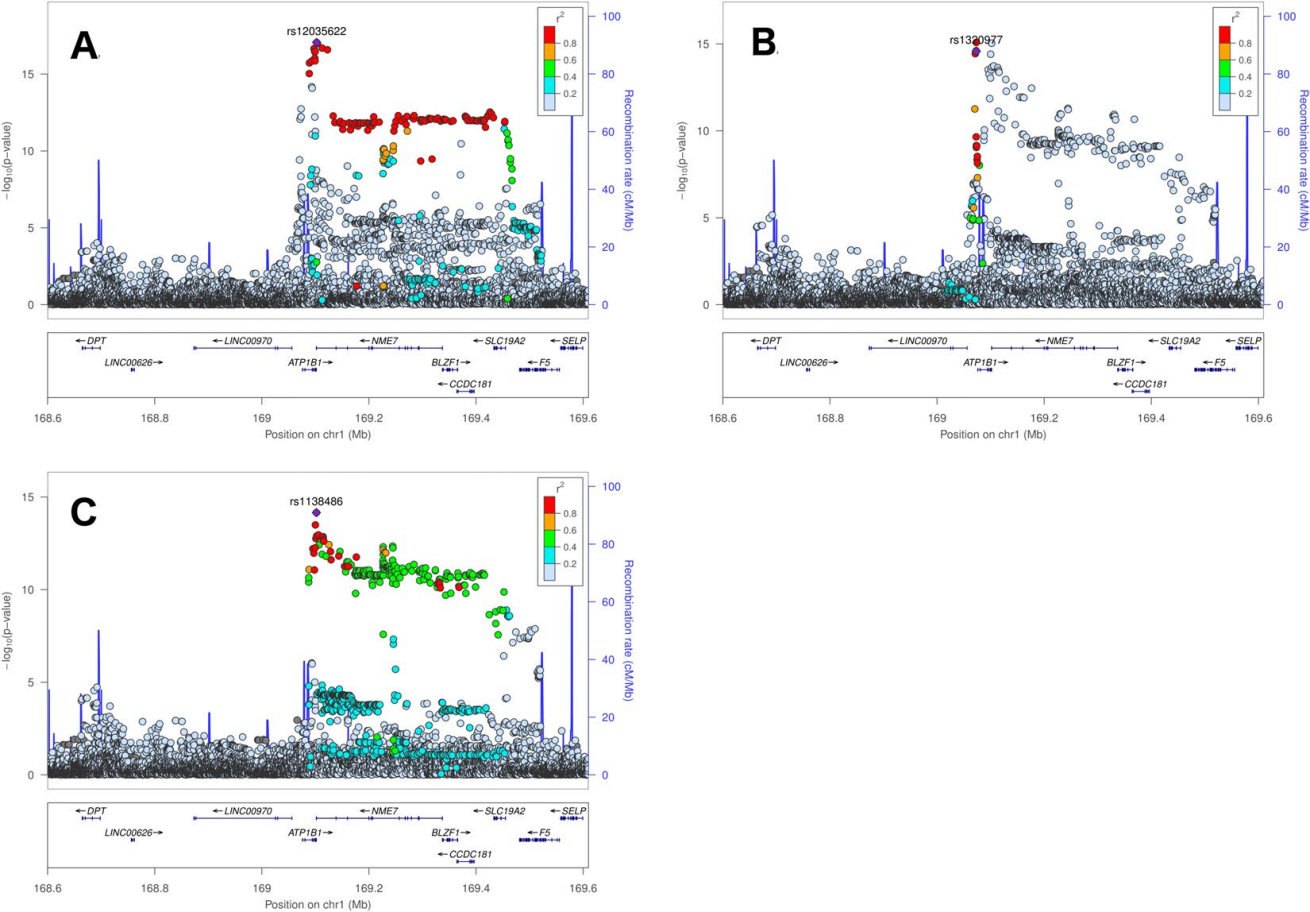
Locus zoom plots of the ATP1B1 gene region providing p-values from the primary (**A**) and conditional analyses (**B**,**C**). The lead SNP in the primary analysis is the previously reported rs12035622 (panel A), the secondary lead SNP after conditioning on rs12035622 is rs1320977 (panel B), and the secondary signal after conditioning on rs12035622 and rs1320977 simultaneously, is rs1138486 (panel C).

**Figure 4:**
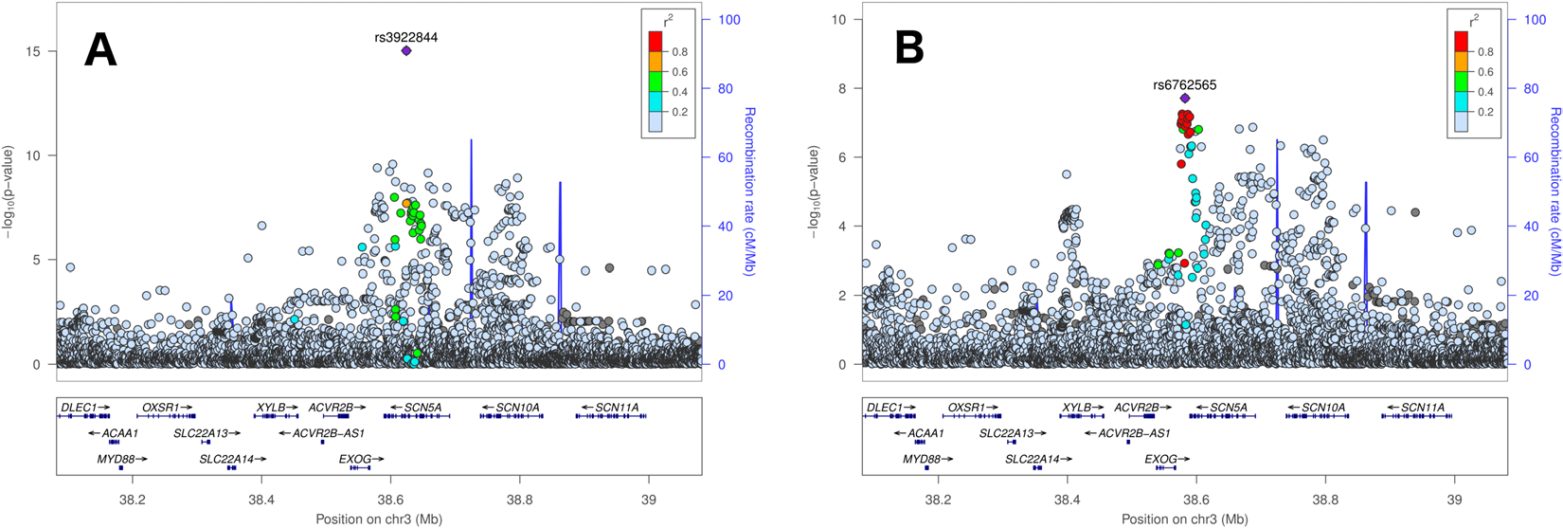
Locus zoom plots of the *SCN5A* gene region providing p-values from the primary (**A**) and conditional analyses (**B**). The lead SNP in the primary analysis is the previously reported rs13922844 (panel A), and the secondary lead SNP after conditioning on rs13922844 is rs6762565 (panel B).

**Figure 5:**
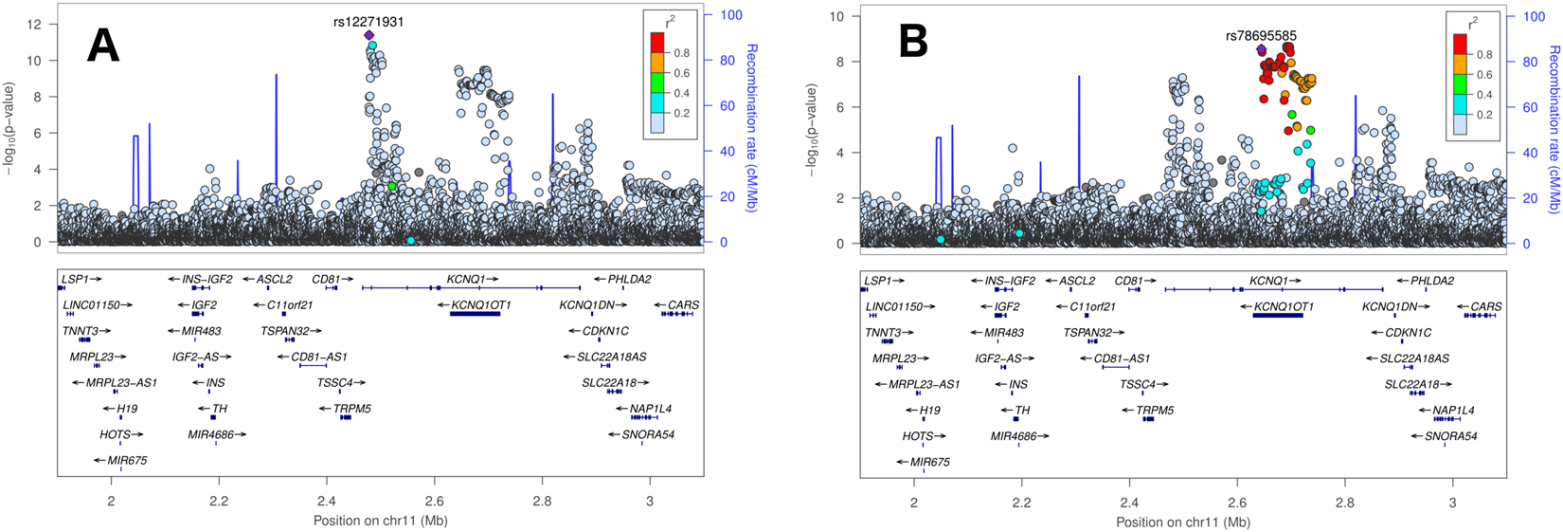
Locus zoom plots of the *KCNQ1* gene region providing p-values from the primary (**A**) and conditional (**B**) analyses. The lead SNP in the primary analysis is the previously reported rs12271931 (panel A), and the secondary lead SNP after conditioning on rs12271931 is rs78695585 (panel B).

Sequential conditional analysis (see Methods) identified four loci with evidence of secondary signals (i.e. SNPs that were uncorrelated with lead SNPs, Table 2): *NOS1AP* (Fig. 2; two secondary signals, rs3934467 and rs73017364), *ATP1B1* (Fig. 3; two secondary signals, rs1320977 and rs1138486), *SCN5A* (Fig. 4; one secondary signal, rs6762565), and *KCNQ1* (Fig. 5); one secondary signal, rs78695585). All six secondary signals at these four loci were correlated (r^2^ > 0.20) with previously identified lead SNPs in the European 1000G super-population (Supplementary Table 5). Wide variation in the linkage disequilibrium (LD) structure for the secondary signals also was observed. For example, SNPs correlated (r^2^ > 0.20; Supplementary Table 5) with the *ATP1B1* lead SNPs and secondary signals spanned ~400 kb (Fig. 3). In contrast, the secondary signals at *NOS1AP, SCN5A*, and *KCNQ1* (Figs 2, 4, and 5) were characterized by fewer correlated SNPs and narrower flanking intervals. (See Supplementary Figure 3 for locus zoom plots for genome wide significant loci without evidence of secondary signals).

**Table 2:** Genome-wide significant secondary SNPs from previously reported regions, identified in meta-analyzed conditional analysis.

Locus	SNP	Chr	Position (hg19)	A1	A2	CAF	β (ms)	Direction of β	SE (ms)	P-val	P_Het_
*NOS1AP*	rs3934467	1	162,182,677	T	C	0.28	1.62	++++	0.24	2.26e–11	0.78
	rs73017364	1	162,184,746	T	C	0.87	1.73	++++	0.31	3.74e–08	0.65
*ATP1B1*	rs1320977	1	169,073,388	A	G	0.15	−2.30	−−−+	0.29	2.61e–15	0.02
	rs1138486	1	169,101,935	T	C	0.14	−2.46	−−−?	0.31	6.98e–15	0.55
*SCN5A*	rs6762565	3	38,582,191	T	C	0.19	−1.65	?−−?	0.29	1.94e–08	0.19
*KCNQ1*	rs78695585	11	2,644,544	A	G	0.04	3.48	++++	0.59	2.82e–09	0.63

Chr: chromosome number. Position: the base pair position in Build 37 (hg19). A1, A2: coded/non-coded alleles. CAF: coded allele frequency. β: effect estimate in ms for the highest associated SNP upon conditional analysis. Direction: the direction of the effect estimates; order is WHI, MESA, HCHS/SOL, and Starr County; ‘?’ means the SNP was not present for a particular study. SE: standard error (ms). P_het_: P-val for Cochran’s Q test of homogeneity among cohorts, for the highest associated SNP upon conditional analysis.

### Generalization analysis

Next we evaluated 34 index SNPs reported as genome-wide significant by the largest QT GWAS published to-date in up to 103,000 European ancestry individuals^19^. A total of 27 of the 34 (79%) previously identified index SNPs generalized to Hispanic/Latinos (r-value < 0.05) (Supplementary Figure 4), with effects similar to those in the original GWAS. Of note, eight of the 27 index SNPs that generalized also were associated with QT in Hispanic/Latinos at genome-wide significant levels (*RNF207, NOS1AP, ATP1B1, SLC8A1, SLC35F1, KCNQ1, LITAF*, and *SETD6*). Among the seven index SNPs that did not generalize at the *KCNJ2*, *C3ORF75*, *GFRA3*, *GMPR*, *CAV1*, *AZIN1*, and *ANKRD9* loci, the consistency in directions of estimated associations between the HCHS/SOL and Arking *et al*.^19^ is higher than what is expected by chance (p-value = 0.01 on a binomial test), suggesting that at least some of the variants that did not generalize are in fact associated with QT in Hispanics/Latinos and that non-generalization was due to lack of power (Supplementary Figure 4 and Supplementary Table 6).

### Bioinformatic characterization

For several of the genome-wide significant SNPs associated, we identified strong experimental evidence for transcriptional activation in heart tissue, including the *ATP1B1*, *TTN*, *SCN5A*, and *KCNH2* loci. Conversely, *SLC35F1*, *SETD6* and *KCNE1* had weaker evidence for transcriptional activation; and *NOS1AP*, *KCNQ1* and *LITAF* had epigenetic marks identifying them as putative enhancers of gene transcription. (See Supplementary Table 7 for additional results of bioinformatic characterization).

## Discussion

In this investigation, the first GWAS of Hispanic/Latinos, we identified 13 loci associated with QT at the genome-wide significant thresholds. Although all genome-wide significant loci were reported in earlier QT GWAS^13,15–17,19–22^, we also identified potential evidence of novel and population-specific SNPs at the *SCN5A* and *KCNE1* loci. Further, we reported several suggestive and biologically plausible loci as promising candidates for future follow-up. Together, our results underscore the utility of extending GWAS to include currently under-represented populations to enable improved characterization of the genomics of complex traits like QT.

The majority of participants included in GWAS to-date, including QT GWAS, are of European descent^27,28^, which limits the relevance of medical genomics globally and fails to leverage human diversity to identify novel loci and improve fine-mapping resolution. Hispanics/Latinos – long understudied in large scale genomics efforts - may be particularly informative for QT GWAS due to an increased prevalence of QT- prolonging and SCD-predisposing obesity and diabetes^25,29^. Indeed, loci common to QT, obesity, and diabetes have been identified (e.g. *KCNQ1*)^30,31^. Further, while studies have reported a decreased SCD incidence in Hispanic/Latinos compared to African Americans or European Americans^32,33^, these discordant observations - consistent with the “Hispanic paradox” of lower cardiovascular disease risk despite higher risk factor levels - may reflect ethnic misclassification, selective migration and incomplete cause of death ascertainment rather than decreased SCD incidence^34–38^. In addition to shouldering a greater burden of QT-increasing risk factors, Hispanic/Latino populations are composed of differing proportions of European, African, and Amerindian ancestry^39^. Therefore, including Hispanics/Latinos in GWAS allows examination of SNPs that may be uncommon, rare, or absent in other populations. For example, the KCNE*1* index SNP rs12626657, which appeared to be population-specific, was monomorphic in European populations, but is common in AMR and ASN populations. Thus, the overarching genetic architecture and risk factor profiles of Hispanic/Latino populations may be uniquely positioned to inform the biology underlying QT prolongation and its downstream consequences, e.g. SCD.

Despite the expected benefits of studying Hispanic/Latinos for mapping novel QT loci, our novel genome-wide significant findings were limited to the identification of two potentially population-specific SNPs at established loci. Interestingly, *SCN5A* lead SNP rs3922844 was identified as the lead SNP in PR interval^40^ and QRS^41^ GWAS in African American populations. Thus, while the same loci may influence QT across global populations, ancestrally specific SNPs also exist. Limited success mapping novel loci may reflect several factors including sample size. Yet, several suggestive and biological plausible loci deserve mention, particularly *AJAP1, CAP2*, and *BVES*. For example, a prior QT GWAS in East Asian populations also reported that SNPs at the *AJAP1* locus, a chromosomal region with few ties to cardiac conduction, were suggestively associated with QT^22^. *CAP2*, located approximately one mega base from the previously described *GMPR* QT locus^19^, also is commonly deleted in 6p22 syndrome, a condition characterized by developmental delays and heart defects^42,43^. Interestingly, *CAP2* murine knockouts developed cardiac conduction defects, leading to sudden cardiac death from complete heart block^44^. Finally, an effort using epigenomic signatures to validate loci suggestively associated with QT^19^ reported that mice homozygous for loss-of-function *BVES* alleles exhibited cardiac conduction and pacemaker defects. Knockdown of *bves* in zebrafish also produced shortening of the action potential duration, a QT correlate^45^.

Clearly *AJAP1, CAP2*, and *BVES* remain suggestive until formal replication is achieved. Yet, it is important to again highlight the wide variation in minor allele frequencies observed across global populations. Thus, in the absence of an independent, large population of Hispanic/Latinos with the requisite genotype and electrocardiographic characterization, future attempts at replication and novel locus identification should consider multi-ethnic populations of European, African, and Amerindian descent given the tri-admixed nature of Hispanic/Latinos populations^39^. Indeed, further advances in genotype arrays designed to capture African and Amerindian-specific content, combined with improved reference panels, will likely enable large trans-ethnic meta-analyses, thereby negating the current practice of race/ethnic-specific analyses. Trans-ethnic GWAS also would be valuable for locus refinement and fine-mapping, given that several loci, including *ATP1B1*, remain prohibitively large in size, making identification of underlying functional variants difficult. Further potentially fruitful avenues of inquiry also could include evaluation of exome or whole-genome sequencing data, given the existence of highly penetrant mutations for QT^46^, which have undergone limited characterization in diverse racial/ethnic populations despite repeated calls for greater diversity in large-scale genomics research^47^.

Despite many strengths, this work had several limitations that deserve consideration. The main limitation of our work is sample size, given that prior QT GWAS in European ancestral populations had sample sizes that for some loci that exceeded 100,000 participants. Yet, we successfully generalized 79% of previously identified loci, despite a considerably smaller sample size. Evidence of population-specific signals and biologically plausible suggestive loci not previously detected by prior large GWAS further underscore the value of examining under-represented populations. Second, generalizability of study results to Hispanic/Latinos is unknown. However, studies such as the HCHS/SOL included large samples of Hispanic/Latino participants from diverse countries of origin, helping to ensure that relatively broad representation was achieved. Finally, similar to a previously published African American QT GWAS^20^, our study participants were predominantly female and obese, with a high prevalence of diabetes. It is unclear how these characteristics, known to affect QT^25,26,48^, might have affected study findings or the ability to compare results across populations with differing characteristics.

In summary, our meta-analysis of four Hispanic/Latino populations generalized a majority of the previously identified QT loci, thereby demonstrating the global relevance of these loci. We also detected novel and potentially population-specific signals, one of which was monomorphic in European populations and another that has been reported in GWAS of other cardiac conduction traits in African Americans, possibly indicating population-specific variation in the genetic architecture underlying QT. Finally, we reported several highly promising and biologically plausible suggestive loci not identified in previous GWAS with substantially larger sample sizes. There is a delicate balance between the use of QT measurements tailored to particular subpopulations versus their generalization to the general population to prevent TdP and/or prescribing drugs that minimize the risk of causing the latter, as pointed by Diemberger *et al*.^49^ and Poluzzi *et al*.^50^. Together, these findings underscore the utility of including genetic data of diverse racial/ethnic groups within GWAS in an attempt to better understand the genetic architecture of complex phenotypes like QT.

## Methods

### Study populations

This meta-analysis included 15,997 participants of Hispanic/Latino descent from the following four studies: the HCHS/SOL (n = 11,932)^51,52^, the Multi-Ethnic Study of Atherosclerosis (MESA, n = 1,431)^53^, Starr County Study (n = 883)^54^, and the WHI (n = 1,751)^55^ (see Supplementary Materials and Methods).

### Electrocardiography

Within each cohort, ECGs were recorded by certified technicians using standard 12-lead apparatus and protocols. In the case of HCHS/SOL, MESA and WHI, the QT duration is the maximum time in ms between the earliest onset of the QRS complex to the latest offset of the T wave among the median QT intervals across all 12 leads (see Supplementary Table 2). Participants with poor quality ECGs, atrial flutter or fibrillation on ECG, intraventricular conduction delay, a paced rhythm, or a QRS duration ≥ 120 were excluded from analysis.

### Genotyping and imputation

Participants were genotyped on either the Affymetrix Genome-Wide Human SNP Array 6.0 (MESA, Starr County, and WHI) or an Illumina custom array that consisted of the Illumina Omni 2.5 M array (HumanOmni 2.5-8v1-1) and ~150,000 custom SNPs selected to include ancestry-informative markers, variants characteristic of Native American populations, previously identified GWAS loci, and other candidate gene polymorphisms (HCHS/SOL)^39^ (Supplementary Table 2). Following study-specific genotype QC (Supplementary Table 2), imputation was performed for approximately 38 million SNPs based on the 1000G phase 1 reference panel^56^.

### Statistical Analysis

A maximum of 17,586,686 imputed SNPs (Supplementary Table 2 for details) were examined for associations with QT under an additive genetic model using linear regression (MESA, Starr County, and WHI) or linear mixed models (HCHS/SOL)^39^. The association of each SNP with QT was adjusted for age, sex, heart rate, ancestral principal components, and study site/region, when appropriate, to maintain consistency with previously published QT GWAS^19^. Associations in the HCHS/SOL study were further adjusted for beta-blocking medication use, a significant predictor of QT in HCHS/SOL, sampling weights, and genetic analysis group^39^.

We excluded SNPs that either mapped to the same base pair position or the same rsid, identified using the UCSC Table browser (https://genome.ucsc.edu/cgi-bin/hgTables). We also excluded SNPs with imputation quality metrics <0.3 or with small effective sample sizes (*effN* < 30), defined within each study for each SNP as: $effN=2\times MAF\times (1-MAF)\times N\times Imputation\,Quality$; where N is the number of participants. Fixed- effects inverse variance meta-analysis was then performed using METAL^57^ on genomically controlled study-specific summary statistics to combine effect estimates (β coefficients) and standard errors (SE). Heterogeneity among studies was assessed by Cochran’ Q test. Complete meta-analysis results are available on dbGAP (https://www.ncbi.nlm.nih.gov/gap) with accession number phs000930.

Genome wide significant associations were defined as SNPs with P-value < 5 × 10^−8^ (Bonferroni correction for ∼10^6^ independent variants). Suggestive associations were those with P-val < 5 × 10^−6^. To identify secondary signals, we performed sequential conditional analyses by adjusting for significant Hispanic/Latino lead SNPs until no remaining genome-wide significant SNPs remained. Population-specific SNPs were defined as SNPs in low LD (r^2^ < 0.20)^58,59^ with previously reported SNPs in the population in which the SNP was discovered [using 1000G Project phase-1^60^ summary results (EUR, AMR, AFR, ASN) and the Application Program Interface (API) in Perl provided by ENSEMBL (http://useast.ensembl.org/info/docs/api/variation/variation_tutorial.html)].

### Generalization

For SNPs previously reported as significantly associated with QT in published GWAS (i.e. P-value < 5 × 10^−8^), we used the approach by Sofer *et al*. to examine evidence for generalization^61^, i.e. the replication of SNP-phenotype associations in a population with different ancestry than the population in which the associations were first identified. Briefly, Sofer *et al*.*’s* approach assigned an r-value to every index SNP, and the generalization null hypothesis testing generalization of the QT index SNPs to Hispanic/Latinos was rejected when the r-value < 0.05, controlling the false discovery rate. For each SNP, we presented confidence intervals of the association effect in the discovery study^19^ alongside confidence intervals of the effect in Hispanic/Latino populations.

### Functional Annotation

We used epigenetic data from the ENCODE^62^ and RoadMap^63^ projects to functionally annotate significant loci (lead SNP, secondary signals, and any SNPs in high LD (r^2^ > 0.80) with lead SNPs or secondary signals in Hispanic/Latinos) using the HaploReg v4.1 on-line resource^64^ and the Chromatin 15-state model, based on ChromHMM provided within the latter. Functional annotation was restricted to heart tissue (fetal heart, right and left atrium and left ventricle). Although the LD pattern used in HaploReg v4.1 is based on the AMR 1000G Phase-1 super-population, the data on ENCODE and RoadMap come from individuals of heterogeneous (or unknown) ancestry (https://docs.google.com/spreadsheets/d/1yikGx4MsO9Ei36b64yOy9Vb6oPC5IBGlFbYEt-N6gOM/edit#gid=15). In addition to the summary of the functional annotation results, Supplementary Table 8 provides biological function and previously known polymorphisms for the 13 genome-wide significant loci associated with QT in Hispanic/Latinos.

## Electronic supplementary material

Supplementary Material
