# Supplementary material for: GWAS of the electrocardiographic QT interval in Hispanics/Latinos generalizes previously identified loci and identifies population-specific signals
Source: Sci Rep. 2017 Dec 6;7:17075. doi: 10.1038/s41598-017-17136-0 (PMC5719082; doi:10.1038/s41598-017-17136-0)
Supplement: Supplementary file 1 — Supplementary Material [file 41598_2017_17136_MOESM1_ESM.pdf]

GWAS of the electrocardiographic QT interval in Hispanics/Latinos generalizes previously identified loci and identifies population-specific signals

Raúl Méndez-Giráldez[1*], Stephanie M. Gogarten[2], Jennifer E. Below[3], Jie Yao[4], Amanda A. Seyerle[1,5], Heather M. Highland[1], Charles Kooperberg[6], Elsayed Z. Soliman[7,8], Jerome I. Rotter[4], Kathleen F Kerr[2], Kelli K Ryckman[9], Kent D. Taylor[4], Lauren E. Petty[10,11], Sanjiv J. Shah[12], Matthew P. Conomos[2], Nona Sotoodehnia[13,14], Susan Cheng[15], Susan R. Heckbert[13,16], Tamar Sofer[2], Xiuqing Guo[4], Eric A. Whitsel[1,17], Henry J. Lin[4,18], Craig L. Hanis[10], Cathy C. Laurie[2], Christy L. Avery[1,19*]

SUPPLEMENTAL MATERIAL

SUPPLEMENTAL TEXT 3
Supplementary Text: Description of Participating Studies 3
SUPPLEMENTAL FIGURES AND TABLES 5
Supplementary Tables 5
Supplementary Figures 22
REFERENCES 27

SUPPLEMENTAL TEXT

Supplementary Text: Description of Participating Studies

The Hispanic Community Health Study/Study of Latinos (HCHS/SOL)

The HCHS/SOL is a multicenter, community-based cohort study of U.S. Hispanic/Latinos (1, 2). Goals of the study are to examine the prevalence of and risk factors for several disorders including heart, lung, blood, and kidney phenotypes. HCHS/SOL investigators sampled 16,415 males and females aged 18-74 years at baseline from four study communities: The Bronx, NY, Chicago, IL, Miami, FL, and San Diego, CA. HCHS/SOL recruitment centers were selected so that the study would include at least 2,000 participants in each of the following designations: Mexican, Puerto Rican, Dominican, Cuban, and Central and South American. A two-stage sampling design was used to recruit participants. The current study includes participants who consented to genotyping.

Multi-Ethnic Study of Atherosclerosis (MESA)

MESA was initiated in 2000 to investigate subclinical cardiovascular disease and the risk factors that predict progression to clinically overt cardiovascular disease (3). The population-based cohort included 6,814 asymptomatic males and females aged 45–84 at study baseline from six field centers (Winston-Salem, NC; St. Paul, MN; Chicago, IL; Los Angeles, CA; New York, NY; Baltimore, MD). MESA investigators enrolled participants of Caucasian (38%), African American (28%), Hispanic (22%) and (12%) Chinese descent. The current study was restricted to Hispanic/Latino MESA participants who gave consent for DNA use. ECGs were measured at study baseline.

Starr County

These data were generated from examinations performed in follow-up from a systematic enumeration of 2507 households from 309 blocks selected randomly from the major population centers of Starr County, Texas. All subjects self-identified as Mexican American. Details of the participant examinations are previously described (4) as is generation, imputation, and quality control analyses of genotype data (5) Echocardiography was among the measures taken; after sample exclusions 582 individuals remained in the analysis of QRS duration, and 680 remained in the analysis of QT interval.

Women's Health Initiative Clinical Trial (WHI CT)

The WHI comprises both randomized clinical trials (CT) and an observational study (OS). This study is limited to WHI CT participants, as ECGs were not collected for WHI OS participants (6). The three WHI clinical trials were designed to allow randomized, controlled evaluation of 1) estrogen with or without progestin treatment, 2) calcium/vitamin D supplementation, and 3) dietary modification on the risk of breast and colorectal cancer, cardiovascular disease, and bone fractures. Between 1993 and 1998, the trials enrolled 68,132 postmenopausal women aged 50–79 years who were followed at 1 of 75 US examination sites (including satellites, remote sites, and their changes in location). Women were ineligible if they had medical conditions predictive of survival time less than 3 years, if they were known to have conditions inconsistent with study participation and adherence, or if they were active participants in another randomized, controlled trial. Those who remained eligible and interested were invited to follow-up examinations at 1, 3, 6, and 9 years. ECGs were measured at study baseline.

SUPPLEMENTAL FIGURES AND TABLES

Supplementary Tables

Supplementary Table 1. Participant-specific characteristics for the four populations involved in our study, WHI, MESA, SOL and Starr County.

	Contributing Studies			
**Characteristic**	**HCHS/SOL**	**MESA**	**Starr County**	**WHI**
N	11,932	1,431	883	1,751
Sex, female %	59.9	51.9	70.8	100.0
Age, years mean (range)	46 (18 – 76)	61 (44 – 84)	53 (32 – 87)	60 (50 - 79)
QT interval, ms, mean (SD)	416 (28)	409 (30)	399 (30)	401 (30)
BMI, kg/m^2, mean	29.8	29.5	32.2	29.4
Hypertension, %	27.3	41.9	42.5	30.4
Diabetes mellitus, %	19.3	17.8	45.6	8.0
Heart rate, bpm, mean	63	63	68	66
Genomic control (λ_{GC})	1.02	1.02	0.98	1.02

BPM: beats per minute; HCSH/SOL: Hispanic Community Health Study / Study of Latinos; MESA: Multi-ethnic Study of Atherosclerosis; MS: milliseconds; SD: standard deviation; WHI: Women Health Initiative.

Supplementary Table 2. ECG and genotype measurement methods for the HCHS/SOL, MESA, Starr County, and WHI studies.

Study	HCHS /SOL	MESA	Starr County	WHI
**ECG Measurements**				
ECG Machine	GE MAC 1200	GE MAC 1200	Nihon Kohden CardioFax ECG-9320A	Marquette MAC PC
ECG Measurement System	GE Marquette 12-SL software	GE Marquette 12-SL software (2001 version)	Nihon Kohden ECAPs software	Marquette 12SL software
**Genotype**				
Array	Illumina HumanOmni2.5-8v1-1 + custom content	Affymetrix Genome-Wide Human SNP Array 6.0 (Affymetrix, Santa Clara, CA)	Affymetrix 1751 (Affymetrix, Santa Clara, CA)	Affymetrix Genome-Wide Human SNP Array 6.0 (Affymetrix, Santa Clara, CA)
Genotype calling software	GenomeStudio v2011.1	Birdseed v1.33	Birdseed and CRLMM concordant calls	Birdseed
SNP call rate genotyping exclusion	<98%	<95%	<90%	≤95%
SNP MAF genotyping exclusion	NA	<1%	<1%	<1%
SNP MAC genotyping exclusion	NA	NA	NA	NA
P HWE genotyping exclusion	<1e-5	<1e-6	<1e-4	<1e-6
MAF imputation exclusion	NA	0.01	<0.05	NA
Imputation quality exclusion	NA	<0.4	<0.8	NA
Imputation software	IMPUTE2	IMPUTE2	IMPUTE2	MaCH v1.0.16
Build used for Imputation	1000 Genomes Phase I release 3 (NCBI build 37 / hg19)	1,000 Genomes Phase I integrated variant set (NCBI build 37 / hg19)	1,000 Genomes Phase I integrated variant set (NCBI build 37 / hg19)	1,000 Genomes Phase I (NCBI Build 37/ hg19)

GWAS statistical analysis software	R/Bioconductor GENESIS package	SNPTEST2	SNPTEST2	ProbAbel
Related Individuals?	Yes	No	Cleaned from analysis with PRIMUS (7, 8)	No
Familial Adjustment method	Kinship coefficients in Mixed model	NA	NA	NA
# SNPs measured	2,232,944	881,666	603,042	934,930
# SNPSs imputed	25,568,744	39×10^6	39×10^6	38×10^6
# SNPs passing QC	17,322,742	8,637,954	5,997,534	8,217,098

Supplementary Table 3. Coded allele frequencies for 13 lead SNPs significantly associated with QT among 15,997 participants of Hispanic/Latino ancestry.

						CAF			
**Locus**	**Lead SNP**	**Chr**	**Position (hg19)**	**A1**	**A2**	**AFR**	**AMR**	**ASN**	**EUR**
RNF207	rs7531322	1	6299823	C	G	0.48	0.70	0.55	0.66
NOS1AP	rs12143842	1	162033890	T	C	0.14	0.26	0.32	0.24
ATP1B1	rs12035622	1	169102340	A	T	0.01	0.22	0.02	0.12
SLC8A1	rs35450971	2	40754314	T	C	0.28	0.23	0.43	0.05
TTN	rs55863869	2	179647546	A	G	0.00	0.11	0.22	0.06
SCN5A	rs3922844	3	38624253	T	C	0.34	0.67	0.86	0.71
SLC35F1	rs2078383	6	118706643	T	C	0.16	0.26	0.25	0.46
KCNH2	rs35760656	7	150658678	A	G	0.35	0.33	0.64	0.22
KCNQ1	rs12271931	11	2478519	A	G	0.99	0.90	1.00	0.88
LITAF	rs735951	16	11693536	A	G	0.43	0.39	0.54	0.44
SETD6	rs185639574	16	58550052	T	G	0.13	0.41	0.36	0.23
PRKCA	rs56152251	17	64280153	A	G	0.52	0.40	0.57	0.45
KCNE1	rs12626657	21	35828173	A	G	0.08	0.18	0.44	0.00

Chr: chromosome. CAF: coded allele frequency. A1, A2: coded and non-coded alleles. 1000G Phase-1 super-populations: AFR, African; AMR, Ad Mixed Americans; ASN, East Asians; and EUR, Europeans.

Supplementary Table 4. Lead SNPs for suggestive loci not previously reported in published QT GWAS estimated among 15,997 participants of Hispanic/Latino ancestry.

SNP	Chr	Position (GRCh37)	A1	A2	CAF	β (ms)	SE (ms)	P-val	Direction	Nearest Gene	CAF			
											AFR	AMR	ASN	EUR
rs11576972	1	4,897,644	C	G	0.15	1.43	0.31	2.80e-06	++++	*AJAP1*	0.35	0.11	0.10	0.19
rs146328487	1	66,506,649	C	G	>0.99	9.70	2.02	1.52e-06	??+?	*PDE4B*	0.00	0.01	0.00	0.00
rs112611436	1	68,600,247	T	C	>0.99	12.42	2.61	1.91e-06	??+?	*LOC100289178*	0.00	0.00	0.00	0.01
rs138291765	1	181,199,691	A	G	0.99	-4.90	1.07	4.68e-06	??-?	*IER5*	0.00	0.01	0.04	0.01
rs77857032	2	149,336,211	A	G	>0.99	-11.80	2.42	1.05e-06	??-?	*MBD5*	0.01	0.00	0.06	0.00
rs976973	2	177,362,096	A	G	0.33	-1.12	0.24	3.66e-06	----	*MIR1246*	0.32	0.64	0.92	0.59
rs10432489	2	181,677,803	A	G	0.75	-1.32	0.27	6.45e-07	----	*UBE2E3*	0.03	0.22	0.22	0.05
rs13413665	2	182,127,663	T	C	0.98	3.36	0.72	2.95e-06	+++?	*MIR4437*	0.06	0.02	0.00	0.02
rs144065589	2	219,802,088	T	G	>0.99	-13.26	2.76	1.51e-06	??-?	*CDK5R2*	0.00	0.00	0.00	0.01
rs141706116	3	75,394,093	A	G	0.01	6.43	1.38	3.18e-06	??+?	*FAM86DP*	0.00	0.00	0.00	0.01
rs79670133	3	183,009,205	T	C	0.02	3.85	0.81	2.23e-06	+?+?	*MCF2L2*	0.00	0.02	0.00	0.03
rs138682672	4	54,664,020	A	G	<0.01	13.16	2.58	3.27e-07	??+?	*RPL21P44*	0.01	0.00	0.00	0.00
rs150851617	4	79,106,410	T	C	>0.99	14.58	3.09	2.34e-06	??+?	*FRAS1*	0.00	0.00	0.00	0.00
rs117824133	4	86,689,576	A	C	<0.01	-14.82	3.09	1.67e-06	??-?	*ARHGAP24*	0.00	0.00	0.02	0.00
rs114950282	5	3,657,281	T	C	0.01	-8.44	1.58	8.51e-08	??-?	*IRX1*	0.03	0.00	0.00	0.00
rs78458156	5	29,892,870	T	C	0.99	6.39	1.33	1.42e-06	??+?	*LOC729862*	0.00	0.01	0.00	0.02
rs190157126	5	50,457,244	A	G	<0.01	-13.50	2.90	3.21e-06	??-?	*ISL1*	0.00	0.01	0.00	0.00
rs1320997	6	17,369,027	A	C	0.07	2.27	0.44	2.69e-07	++++	*CAP2*	0.01	0.04	0.21	0.01
rs151299662	6	76,986,551	T	C	0.01	9.70	1.96	7.45e-07	??+?	*IMPG1*	0.00	0.01	0.00	0.01
rs405956	6	105,554,355	A	G	0.85	-1.49	0.31	2.10e-06	---?	*BVES*	0.43	0.87	0.87	0.89
rs188964420	6	127,025,773	T	G	>0.99	-12.33	2.59	1.93e-06	??-?	*CENPW*	0.01	0.00	0.00	0.00
rs190758711	6	127,253,075	C	G	<0.01	12.03	2.62	4.22e-06	??+?	*RSPO3*	0.00	0.00	0.00	0.00
rs192338477	6	140,744,550	T	C	>0.99	11.50	2.39	1.56e-06	??+?	*MIR3668*	0.01	0.00	0.00	0.00
rs73700722	7	68,798,601	A	G	0.99	-7.08	1.35	1.45e-07	??-?	*AUTS2*	0.05	0.00	0.00	0.00

rs6975094	7	133,179,240	A	G	>0.99	-9.99	1.98	4.65e-07	??-?	*EXOC4*	0.97	1.00	1.00	1.00
rs6989175	8	25,108,235	A	C	0.30	1.25	0.24	2.56e-07	+++?	*DOCK5*	0.47	0.28	0.15	0.12
rs144230162	8	72,639,323	A	G	0.99	8.60	1.72	5.60e-07	??+?	*MSC*	0.00	0.01	0.00	0.00
rs138693040	8	133,320,795	T	G	>0.99	11.99	2.49	1.53e-06	??+?	*KCNQ3*	0.01	0.00	0.00	0.00
rs181483657	9	139,664,092	C	G	0.05	26.30	5.08	2.22e-07	???+	*LCN15*	0.15	0.06	0.03	0.05
rs146672695	11	68,399,485	A	C	0.05	2.39	0.51	2.29e-06	+++-	*PPP6R3*	0.05	0.07	0.01	0.04
rs7945404	11	121,206,662	T	C	0.92	1.91	0.42	4.54e-06	-+++	*SC5DL*	0.03	0.10	0.00	0.17
rs7131987	12	29,406,488	T	G	0.32	-1.08	0.23	2.24e-06	----	*FAR2*	0.66	0.29	0.38	0.35
rs114194742	14	105,281,925	T	C	<0.01	14.52	3.09	2.63e-06	??+?	*MGC23270*	0.00	0.00	0.00	0.00
rs7201929	16	28,871,966	T	C	0.72	1.20	0.24	5.05e-07	+++?	*SH2B1*	0.29	0.31	0.45	0.23
rs147089336	16	73,352,629	A	G	<0.01	9.58	2.09	4.71e-06	??+?	*LOC100506172*	0.02	0.00	0.00	0.00
rs9900062	17	62,739,574	A	G	0.71	-1.15	0.25	4.77e-06	----	*PLEKHM1P*	0.90	0.68	0.81	0.76
rs12956470	18	6,477,869	A	G	0.17	1.37	0.29	2.67e-06	++++	*L3MBTL4*	0.26	0.17	0.00	0.19
rs12604566	18	70,630,706	T	C	0.61	3.30	0.66	6.79e-07	?+??	*NETO1*	0.66	0.66	0.55	0.76
rs144778694	19	18,916,863	A	G	0.07	-2.45	0.52	2.63e-06	?---	*COMP*	0.31	0.06	0.06	0.02
rs34672598	20	7,884,260	T	C	0.87	2.10	0.45	3.04e-06	??++	*HAO1*	0.03	0.14	0.22	0.09
rs2298679	21	35,247,460	T	C	0.20	-1.38	0.28	7.47e-07	----	*ITSN1*	0.70	0.12	0.14	0.21
rs141878091	22	45,937,021	A	G	<0.01	-10.73	2.29	2.91e-06	??-?	*FBLN1*	0.00	0.01	0.00	0.00

SNP: single nucleotide polymorphism. Chr: chromosome. A1, A2: coded and non-coded alleles. CAF: coded allele frequency. β: effect estimates in ms. SE: standard error for the effect estimate in ms. Direction: the signs of the effect estimates, ordered as WHI, MESA, HCHS/SOL and Starr County. CAF for the 4 super populations in 1000G project Phase-1: Africans (AFR), Ad Mixed Americans (AMR), East Asians (ASN) and Europeans (EUR).

Supplementary Table 5. Comparison between previous published QT GWAS and the 19 genome-wide significant and secondary signals in 15,997 participants of Hispanic/Latino ancestry.

Locus	Chr	GWAS Index SNP	GWAS Position (GRCh38)	GWAS Discovery Population	Hispanic/Latino Lead SNP	Hispanic/ Latino Position (GRCh38)	r^2 EUR	r^2 AMR	r^2 AFR	r^2 ASN
RNF207	1	rs2273042	6,089,062	EUR	rs7531322	6,239,763	<0.05	<0.05	<0.05	<0.05
	1	rs846111	6,219,310	EUR	rs7531322	6,239,763	0.57	0.55	<0.05	0.18
NOS1AP	1	rs1415259	162,115,519	IND	rs12143842	162,064,100	0.41	0.35	<0.05	0.12
	1	rs10494366	162,115,895	EUR	rs12143842	162,064,100	0.40	0.35	<0.05	0.12
	1	rs16857031	162,143,120	EUR	rs12143842	162,064,100	<0.05	0.07	0.17	0.12
	1	rs12029454	162,163,327	EUR	rs12143842	162,064,100	0.13	0.09	<0.05	0.18
	1	rs17457880	162,198,364	EUR	rs12143842	162,064,100	<0.05	<0.05	<0.05	<0.05
	1	rs4657172	162,209,842	EUR	rs12143842	162,064,100	<0.05	<0.05	<0.05	<0.05
	1	rs3934467	162,212,887	EUR	rs12143842	162,064,100	0.06	0.08	<0.05	0.18
	1	rs7545047	162,221,313	EUR	rs12143842	162,064,100	<0.05	<0.05	<0.05	<0.05
	1	rs4657175	162,225,948	KOR	rs12143842	162,064,100	0.06	0.08	<0.05	0.18
	1	rs4657178	162,240,820	EUR	rs12143842	162,064,100	<0.05	<0.05	<0.05	0.09
	1	rs17460657	162,292,036	EUR	rs12143842	162,064,100	<0.05	<0.05	<0.05	<0.05
	1	rs347272	162,348,708	EUR	rs12143842	162,064,100	0.05	0.08	<0.05	<0.05
	1	rs164133	162,411,498	EUR	rs12143842	162,064,100	<0.05	<0.05	<0.05	<0.05
	1	rs12143842	162,064,100	EUR/JPN	rs3934467	162,212,887	0.06	0.08	<0.05	0.18
	1	rs1415259	162,115,519	IND	rs3934467	162,212,887	0.09	0.06	<0.05	<0.05
	1	rs10494366	162,115,895	EUR	rs3934467	162,212,887	0.09	0.06	<0.05	<0.05
	1	rs16857031	162,143,120	EUR	rs3934467	162,212,887	0.07	<0.05	<0.05	0.07
	1	rs12029454	162,163,327	EUR	rs3934467	162,212,887	0.58	0.52	0.76	0.97
	1	rs17457880	162,198,364	EUR	rs3934467	162,212,887	<0.05	<0.05	<0.05	<0.05
	1	rs4657172	162,209,842	EUR	rs3934467	162,212,887	0.05	0.06	0.19	0.07
	1	rs7545047	162,221,313	EUR	rs3934467	162,212,887	<0.05	<0.05	<0.05	<0.05
	1	rs4657175	162,225,948	KOR	rs3934467	162,212,887	0.99	0.93	0.90	0.98

	1	rs4657178	162,240,820	EUR	rs3934467	162,212,887	0.77	0.56	<0.05	0.49
	1	rs17460657	162,292,036	EUR	rs3934467	162,212,887	<0.05	<0.05	<0.05	<0.05
	1	rs347272	162,348,708	EUR	rs3934467	162,212,887	0.15	0.07	<0.05	<0.05
	1	rs164133	162,411,498	EUR	rs3934467	162,212,887	<0.05	<0.05	<0.05	<0.05
	1	rs12143842	162,064,100	EUR/JPN	rs73017364	162,214,956	<0.05	<0.05	<0.05	<0.05
	1	rs1415259	162,115,519	IND	rs73017364	162,214,956	<0.05	<0.05	<0.05	<0.05
	1	rs10494366	162,115,895	EUR	rs73017364	162,214,956	<0.05	<0.05	<0.05	<0.05
	1	rs16857031	162,143,120	EUR	rs73017364	162,214,956	<0.05	<0.05	<0.05	<0.05
	1	rs12029454	162,163,327	EUR	rs73017364	162,214,956	<0.05	<0.05	0.11	0.07
	1	rs17457880	162,198,364	EUR	rs73017364	162,214,956	<0.05	<0.05	<0.05	<0.05
	1	rs4657172	162,209,842	EUR	rs73017364	162,214,956	0.74	0.59	0.12	1.00
	1	rs3934467	162,212,887	EUR	rs73017364	162,214,956	<0.05	0.06	0.11	0.07
	1	rs7545047	162,221,313	EUR	rs73017364	162,214,956	0.28	0.05	<0.05	<0.05
	1	rs4657175	162,225,948	KOR	rs73017364	162,214,956	<0.05	0.06	0.10	0.07
	1	rs4657178	162,240,820	EUR	rs73017364	162,214,956	<0.05	0.06	0.06	0.14
	1	rs17460657	162,292,036	EUR	rs73017364	162,214,956	<0.05	<0.05	<0.05	<0.05
	1	rs347272	162,348,708	EUR	rs73017364	162,214,956	<0.05	<0.05	<0.05	<0.05
	1	rs164133	162,411,498	EUR	rs73017364	162,214,956	<0.05	<0.05	<0.05	<0.05
ATP1B1	1	rs545833	168,720,702	EUR	rs1320977	169,104,150	<0.05	<0.05	<0.05	<0.05
	1	rs12061601	169,101,212	EUR	rs1320977	169,104,150	0.96	0.79	1.00	0.30
	1	rs1320976	169,104,108	AA	rs1320977	169,104,150	1.00	1.00	1.00	0.99
	1	rs10919070	169,129,799	EUR	rs1320977	169,104,150	<0.05	<0.05	<0.05	<0.05
	1	rs10919071	169,130,245	EUR	rs1320977	169,104,150	<0.05	<0.05	<0.05	<0.05
	1	rs12079745	169,131,822	EUR	rs1320977	169,104,150	<0.05	<0.05	<0.05	<0.05
	1	rs1983546	169,476,945	EUR	rs1320977	169,104,150	<0.05	<0.05	<0.05	<0.05
	1	rs545833	168,720,702	EUR	rs1138486	169,132,697	<0.05	<0.05	<0.05	<0.05
	1	rs12061601	169,101,212	EUR	rs1138486	169,132,697	<0.05	<0.05	<0.05	<0.05
	1	rs1320976	169,104,108	AA	rs1138486	169,132,697	<0.05	<0.05	<0.05	<0.05
	1	rs10919070	169,129,799	EUR	rs1138486	169,132,697	<0.05	<0.05	<0.05	0.07

	1	rs10919071	169,130,245	EUR	rs1138486	169,132,697	<0.05	<0.05	<0.05	0.07
	1	rs12079745	169,131,822	EUR	rs1138486	169,132,697	0.98	0.95	0.62	0.99
	1	rs1983546	169,476,945	EUR	rs1138486	169,132,697	0.10	0.11	0.06	0.14
	1	rs545833	168,720,702	EUR	rs12035622	169,133,102	<0.05	<0.05	<0.05	<0.05
	1	rs12061601	169,101,212	EUR	rs12035622	169,133,102	<0.05	<0.05	<0.05	<0.05
	1	rs1320976	169,104,108	AA	rs12035622	169,133,102	<0.05	<0.05	<0.05	<0.05
	1	rs10919070	169,129,799	EUR	rs12035622	169,133,102	0.93	0.98	1.00	1.00
	1	rs10919071	169,130,245	EUR	rs12035622	169,133,102	0.94	0.98	1.00	1.00
	1	rs12079745	169,131,822	EUR	rs12035622	169,133,102	<0.05	<0.05	<0.05	0.07
	1	rs1983546	169,476,945	EUR	rs12035622	169,133,102	0.12	0.17	<0.05	<0.05
SLC8A1	2	rs6544311	40,126,137	EUR	rs35450971	40,527,174	<0.05	<0.05	<0.05	<0.05
	2	rs12997023	40,525,842	EUR	rs35450971	40,527,174	0.64	0.65	0.34	0.99
	2	rs13017846	40,530,651	KOR	rs35450971	40,527,174	0.64	0.63	0.21	0.95
TTN	2	rs7580640	178,383,693	EUR	rs55863869	178,782,819	<0.05	<0.05	<0.05	<0.05
	2	rs12476289	178,777,248	EUR	rs55863869	178,782,819	0.83	1.00	1.00	0.95
	2	rs7561149	178,825,129	EUR	rs55863869	178,782,819	<0.05	<0.05	<0.05	<0.05
SCN5A	3	rs11129795	38,547,672	EUR	rs6762565	38,540,700	0.99	0.97	0.92	0.98
	3	rs12053903	38,551,902	EUR	rs6762565	38,540,700	0.59	0.28	<0.05	0.09
	3	rs6793245	38,557,546	EUR	rs6762565	38,540,700	0.61	0.43	0.08	0.17
	3	rs11708996	38,592,432	EUR	rs6762565	38,540,700	0.11	0.13	<0.05	<0.05
	3	rs11710077	38,616,408	EUR	rs6762565	38,540,700	<0.05	<0.05	<0.05	<0.05
	3	rs6599234	38,673,809	EUR	rs6762565	38,540,700	<0.05	<0.05	<0.05	<0.05
	3	rs6801957	38,725,824	EUR	rs6762565	38,540,700	<0.05	<0.05	<0.05	<0.05
	3	rs11129795	38,547,672	EUR	rs3922844	38,582,762	<0.05	<0.05	<0.05	<0.05
	3	rs12053903	38,551,902	EUR	rs3922844	38,582,762	<0.05	<0.05	<0.05	<0.05
	3	rs6793245	38,557,546	EUR	rs3922844	38,582,762	<0.05	<0.05	<0.05	<0.05
	3	rs11708996	38,592,432	EUR	rs3922844	38,582,762	0.06	0.09	<0.05	<0.05
	3	rs11710077	38,616,408	EUR	rs3922844	38,582,762	0.16	0.16	<0.05	0.31
	3	rs6599234	38,673,809	EUR	rs3922844	38,582,762	<0.05	<0.05	<0.05	<0.05

	3	rs6801957	38,725,824	EUR	rs3922844	38,582,762	<0.05	<0.05	<0.05	<0.05
SLC35F1	6	rs457162	118,214,820	EUR	rs2078383	118,385,480	<0.05	<0.05	<0.05	<0.05
	6	rs12210733	118,331,912	EUR	rs2078383	118,385,480	<0.05	<0.05	<0.05	<0.05
	6	rs12210810	118,332,041	EUR	rs2078383	118,385,480	<0.05	<0.05	<0.05	<0.05
	6	rs11153730	118,346,359	EUR	rs2078383	118,385,480	0.84	0.68	0.25	0.63
	6	rs11970286	118,359,211	EUR	rs2078383	118,385,480	0.97	0.93	0.22	0.74
	6	rs11752626	118,467,489	EUR	rs2078383	118,385,480	0.97	0.89	0.93	0.99
	6	rs11756438	118,672,469	EUR	rs2078383	118,385,480	0.78	0.65	0.29	0.80
	6	rs3902035	118,679,069	EUR	rs2078383	118,385,480	0.06	<0.05	<0.05	<0.05
	6	rs9489510	118,722,735	EUR	rs2078383	118,385,480	<0.05	<0.05	<0.05	0.21
KCNH2	7	rs2968864	150,925,074	EUR	rs35760656	150,961,590	0.09	0.13	<0.05	<0.05
	7	rs2968863	150,926,049	EUR	rs35760656	150,961,590	0.09	0.13	<0.05	<0.05
	7	rs4725982	150,940,775	IND	rs35760656	150,961,590	0.94	0.78	0.55	0.62
	7	rs2072413	150,950,881	EUR	rs35760656	150,961,590	<0.05	<0.05	<0.05	<0.05
	7	rs3807375	150,970,122	EUR	rs35760656	150,961,590	0.52	0.47	0.10	0.53
	7	rs3778872	150,972,888	AA	rs35760656	150,961,590	0.69	0.23	0.52	0.20
KCNQ1	11	rs2301696	2,405,754	EUR	rs12271931	2,457,289	<0.05	<0.05	<0.05	<0.05
	11	rs2074238	2,463,573	EUR	rs12271931	2,457,289	0.38	0.41	0.08	<0.05
	11	rs16928297	2,464,890	AA	rs12271931	2,457,289	<0.05	0.05	<0.05	<0.05
	11	rs7122937	2,465,320	EUR	rs12271931	2,457,289	<0.05	<0.05	<0.05	<0.05
	11	rs12296050	2,468,112	EUR	rs12271931	2,457,289	<0.05	0.05	<0.05	<0.05
	11	rs12576239	2,481,089	EUR	rs12271931	2,457,289	<0.05	<0.05	<0.05	<0.05
	11	rs7947981	2,605,104	JPN	rs12271931	2,457,289	<0.05	<0.05	<0.05	<0.05
	11	rs231906	2,731,379	AA	rs12271931	2,457,289	<0.05	<0.05	<0.05	<0.05
	11	rs2301696	2,405,754	EUR	rs78695585	2,623,314	<0.05	<0.05	<0.05	<0.05
	11	rs2074238	2,463,573	EUR	rs78695585	2,623,314	<0.05	<0.05	<0.05	<0.05
	11	rs16928297	2,464,890	AA	rs78695585	2,623,314	<0.05	<0.05	<0.05	<0.05
	11	rs7122937	2,465,320	EUR	rs78695585	2,623,314	<0.05	<0.05	<0.05	<0.05
	11	rs12296050	2,468,112	EUR	rs78695585	2,623,314	<0.05	<0.05	<0.05	<0.05

	11	rs12576239	2,481,089	EUR	rs78695585	2,623,314	<0.05	<0.05	<0.05	<0.05
	11	rs7947981	2,605,104	JPN	rs78695585	2,623,314	0.52	<0.05	<0.05	0.26
	11	rs231906	2,731,379	AA	rs78695585	2,623,314	<0.05	<0.05	<0.05	<0.05
LITAF	16	rs12930096	11,576,902	EUR	rs735951	11,599,680	0.11	<0.05	0.10	<0.05
	16	rs8049607	11,597,897	EUR/AA	rs735951	11,599,680	0.86	0.89	0.63	0.90
	16	rs12444261	11,640,786	EUR	rs735951	11,599,680	0.13	<0.05	<0.05	<0.05
GINS3	16	rs4784934	58,426,022	EUR	rs185639574	58,516,148	<0.05	<0.05	<0.05	<0.05
	16	rs37060	58,532,400	EUR	rs185639574	58,516,148	0.57	0.67	0.76	0.79
	16	rs37062	58,533,334	EUR	rs185639574	58,516,148	0.58	0.67	0.65	0.79
	16	rs246196	58,540,349	EUR	rs185639574	58,516,148	0.60	0.67	0.76	0.78
	16	rs7188697	58,588,274	EUR	rs185639574	58,516,148	0.56	0.65	0.73	0.78
PRKCA	17	rs9892651	66,307,675	EUR	rs56152251	66,284,035	0.65	0.81	0.28	0.97
KCNE1	21	rs1805128	34,449,382	EUR	rs12626657	34,455,875	<0.05	<0.05	<0.05	<0.05

Chr: chromosome number. EUR, Europeans; AMR, Americans; AFR. Africans; ASN, East Asians. EUR. Abbreviations regarding the discovery population: AA African American; JPN, Japanese; KOR, Korean; and IND, Asian Indians.

Supplementary Table 6. Summary statistics for 24 loci previously reported QT GWAS (9) not identified as genome-wide significant in 15,997 participants of Hispanic/Latino ancestry.

							Previous GWAS			Hispanic/Latino GWAS		
Author/Date	Chr	Position (GRCh38)	Nearest Gene	SNP	Coded	Non-coded	CAF	Effect (ms)	P-val	CAF	Effect (ms)	P-val
Arking 2014	1	23,383,982	*TCEA3*	rs2298632	T	C	0.50	0.70	1.00e-14	0.46	1.15	1.85e-07
Arking 2014	2	173,877,880	*SP3*	rs938291	G	C	0.39	0.53	6.00e-10	0.47	0.54	1.30e-02
Arking 2014	2	200,295,976	*SPATS2L*	rs295140	T	C	0.42	0.61	4.00e-13	0.55	0.52	1.78e-02
Arking 2014	3	47,502,513	*C3ORF75*	rs17784882	A	C	0.40	-0.54	3.00e-08	0.40	-0.35	1.06e-01
Arking 2014	4	71,272,499	*SLC4A4*	rs2363719	A	G	0.11	0.97	8.00e-10	0.07	1.19	4.15e-03
Arking 2014	4	94,105,283	*SMARCAD1*	rs3857067	A	T	0.46	-0.51	1.00e-09	0.64	-0.49	3.12e-02
Arking 2014	5	138,238,036	*GFRA3*	rs10040989	A	G	0.13	-0.85	5.00e-11	0.08	-0.68	9.57e-02
Arking 2014	6	16,294,491	*GMPR*	rs7765828	G	C	0.40	0.55	3.00e-10	0.44	0.45	4.12e-02
Arking 2014	7	116,560,038	*CAV1*	rs9920	C	T	0.09	0.79	3.00e-08	0.93	0.74	8.33e-02
Arking 2014	8	70,277,107	*NCOA2*	rs16936870	A	T	0.10	0.99	1.00e-09	0.09	1.12	2.35e-03
Arking 2014	8	97,838,102	*LAPTM4B*	rs11779860	T	C	0.47	0.61	2.00e-10	0.73	0.65	7.50e-03
Arking 2014	8	102,920,617	*AZIN1*	rs1961102	T	C	0.33	0.57	3.00e-09	0.32	0.35	1.25e-01
Arking 2014	10	102,290,249	*GBF1*	rs2485376	A	G	0.39	-0.56	3.00e-08	0.39	-0.83	1.44e-04
Arking 2014	11	61,842,278	*FADS2*	rs174583	T	C	0.34	-0.57	8.00e-11	0.57	-1.34	3.51e-02
Arking 2014	12	110,285,398	*ATP2A2*	rs3026445	T	C	0.36	-0.62	3.00e-12	0.57	-0.88	4.69e-05
Marroni 2009	13	47,588,423	*LOC105370195 - LOC105370196*	rs2478333	A	C	0.33	0.17	4.00e-08	0.23	-0.23	3.82e-01
Arking 2014	13	73,938,985	*KLF12*	rs728926	T	C	0.36	0.57	2.00e-08	0.32	0.73	1.45e-03
Arking 2014	14	102,508,662	*ANKRD9*	rs2273905	T	C	0.36	0.61	4.00e-11	0.25	0.31	2.12e-01
Arking 2014	15	50,552,821	*USP50, TRPM7*	rs3105593	T	C	0.45	0.66	3.00e-12	0.53	0.76	4.15e-04
Arking 2014	16	3,823,641	*CREBBP*	rs1296720	C	A	0.20	0.83	4.00e-10	0.88	0.77	2.78e-02
Arking 2014	16	14,301,575	*MKL2*	rs246185	C	T	0.34	0.72	3.00e-13	0.71	0.60	1.25e-02

Newton-Cheh 2009	17	34,997,363	*LIG3*	rs2074518	T	C	0.46	-1.05	6.00e-12	0.39	-0.58	8.82e-03
Arking 2014	17	35,004,556	*LIG3*	rs1052536	C	T	0.53	0.98	6.00e-25	0.38	0.59	7.51e-03
Arking 2014	17	70,434,852	*KCNJ2*	rs1396515	C	G	0.52	-0.98	2.00e-25	0.58	-0.43	4.63e-02

Chr: chromosome number. Position: base pair position in Build 38 (GRCh38). Nearest Gene: closest gene in sequence to the SNP. CAF: coded allele frequency. Effect: effect estimates in ms. GWAS, genome-wide association study.

Supplementary Table 7. Summary of the functional annotation using HaploReg v4.1 (10) on the 13 genome-wide significant loci. Only the loci with functional annotation in heart tissues are shown.

Locus	SNPs	Regulatory Motif	Nb motifs	Tissues
NOS1AP	7	Enhancers	16	RV;LV;FH;RA
ATP1B1	22	Active TSS	6	RA;LV;RV
		Enhancers	22	RA;LV;FH;RV
		Flanking Active TSS	4	RV;FH;LV
		Genic enhancers	6	RV;LV;FH
TTN	23	Active TSS	6	RV;RA;LV
		Enhancers	5	RV;FH;RA
		Genic enhancers	20	LV;FH
		Transcription at gene 5' and 3'	3	FH;RV
SCN5A	18	Enhancers	42	RV;LV;FH;RA
		Flanking Active TSS	2	FH
		Genic enhancers	1	RV
SLC35F1	2	Active TSS	3	RV;LV;RA
		Enhancers	1	FH
KCNH2	11	Enhancers	24	RV;RA;FH;LV
		Flanking Active TSS	2	FH
		Genic enhancers	3	RV
KCNQ1	12	Enhancers	32	RV;LV;FH;RA
SETD6	1	Active TSS	3	LV;RA;RV
LITAF	2	Enhancers	2	RV
KCNE1	1	Enhancers	3	LV;RA;RV
		Flanking Active TSS	1	FH

SNPs: number of SNPs in LD ($r^2 > 0.8$) with any of the genome-wide associated SNPs in Table 1 or any of their secondary signals in Table 2, in Hispanic/Latinos populations. Regulatory Motif: the chromatin state as defined by the 15-state model in ChromHMM algorithm (11), TSS is Transcription Star Site. Tissues: RV, Right Ventricle; LV, Left Ventricle; RA, Right Atrium; FH, Fetal Heart.

Supplementary Table 8. Polymorphisms known at the loci with SNPs associated genome-wide significantly to QT genome-wide significantly to QT in 15,997 participants of Hispanic/Latino ancestry.

Locus	Variant type	Protein	Function	References
RNF207	missense(3), intronic(1)	Ring Finger Protein 207	*RNF207* (Ring Finger Protein 207) is a Protein Coding gene. GO annotations related to this gene include ion channel binding and Hsp70 protein binding.	(12-14)
NOS1AP	intronic(17), intergenic(7)	Nitric Oxide Synthase 1 (Neuronal) Adaptor Protein	Diseases associated with *NOS1AP* include Long QT Syndrome 1 and Schizophrenia. Among its related pathways are NOS Signaling at Neuronal Synapses and SIDS Susceptibility Pathways. GO annotations related to this gene include nitric-oxide synthase binding.	(12-21)
ATP1B1	intronic(5), upstream_gene(2), 3_prime_UTR(1)	Sodium-Potassium ATPase Subunit Beta 1 (Non-Catalytic)	Diseases associated with *ATP1B1* include Thyrotoxic Periodic Paralysis and Megalencephalic Leukoencephalopathy With Subcortical Cysts. Among its related pathways are Myometrial Relaxation and Contraction Pathways and cAMP signaling pathway. GO annotations related to this gene include ATPase binding and ATPase activator activity.	(13, 14, 19, 20)
SLC8A1	intronic (3)	SLC8A1 Solute Carrier Family 8 (Sodium/Calcium Exchanger), Member 1	Rapidly transports Ca(2+) during excitation-contraction coupling. Ca(2+) is extruded from the cell during relaxation so as to prevent overloading of intracellular stores.	(15, 18)
TTN	intronic (1), missense(1)	Titin	Diseases associated with *TTN* include Cardiomyopathy, Dilated, 1G and Cardiomyopathy, Familial Hypertrophic, 9. Among its related pathways are Platelet activation, signaling and aggregation and Hypertrophic cardiomyopathy (HCM). GO annotations related to this gene include nucleic acid binding and identical protein binding.	(14, 15)
SCN5A	intronic (5), intergenic(1), downstream_gene (1)	Sodium Channel, Voltage Gated, Type V Alpha Subunit	Diseases associated with *SCN5A* include Long QT Syndrome-3 and Brugada Syndrome 1. Among its related pathways are Neuropathic Pain-Signaling in Dorsal Horn Neurons and Activation of cAMP-Dependent PKA. GO annotations related to this gene include protein kinase binding and ubiquitin protein ligase binding.	(12-14)
SLC35F1	intronic(3),intergen ic(3)	Solute Carrier Family 35, Member F1	Putative solute transporter	(12, 16, 17)

KCNH2	intronic (4), intergenic(2), downstream_gene (2)	Potassium Channel, Voltage Gated Eag Related Subfamily H, Member 2	Diseases associated with *KCNH2* include Long QT Syndrome 2 and Short QT Syndrome 1. Among its related pathways are Glucose / Energy Metabolism and Potassium Channels. GO annotations related to this gene include protein homodimerization activity and signal transducer activity.	(12-14, 17, 19, 21)
KCNQ1	intronic (10)	Potassium Channel, Voltage Gated KQT-Like Subfamily Q, Member 1	Diseases associated with *KCNQ1* include Long QT Syndrome 1 and Jervell And Lange-Nielsen Syndrome. Among its related pathways are Potassium Channels and Transmission across Chemical Synapses. GO annotations related to this gene include calmodulin binding and ion channel binding.	(12-14, 17, 19, 20, 22)
LITAF	intronic(5), upstream_gene(1)	Lipopolysaccharide-Induced TNF Factor	Diseases associated with *LITAF* include Charcot-Marie-Tooth Disease, Type 1C and Tooth Disease. Among its related pathways are NF-kappaB Signaling and Lysosome. GO annotations related to this gene include signal transducer activity and WW domain binding.	(13, 14, 19, 23)
SETD6[(*)]	NA	SET Domain-Containing Protein 6	GO annotations related to this gene include NF-kappaB binding and protein-lysine N-methyltransferase activity.	NA
CNOT1[(*)]	intronic(4),splice_region(1)	CCR4-NOT Transcription Complex, Subunit 1	Diseases associated with *CNOT1* include Iritis. Among its related pathways are Gene Expression and Deadenylation-dependent mRNA decay. GO annotations related to this gene include poly(A) RNA binding and protein domain specific binding.	(13, 14, 17, 18, 23)
PRKCA	intronic (1)	Protein Kinase C, Alpha	Diseases associated with *PRKCA* include Glioma and Glioblastoma. Among its related pathways are Development VEGF signaling via *VEGFR2* - generic cascades and IL-2 Pathway. GO annotations related to this gene include transferase activity, transferring phosphorus-containing groups and protein tyrosine kinase activity.	(14)
KCNE1	intronic(1), missense(1)	Potassium Channel, Voltage Gated Subfamily E Regulatory Beta Subunit 1	Diseases associated with *KCNE1* include Long QT Syndrome 5 and Jervell And Lange-Nielsen Syndrome 2. Among its related pathways are Antiarrhythmic Pathway, Pharmacodynamics and Phase 1 - inactivation of fast Na+ channels. GO annotations related to this gene include voltage-gated potassium channel activity and delayed rectifier potassium channel activity.	(14, 17)

*****The *SETD6* (SET Domain-Containing Protein 6) locus, a Protein-lysine N-methyltransferase required for the maintenance of the embryonic cells for which we have a significant hit, has not been associated to QT interval. Although there are several variants associated to *CNOT1* (CCR4-NOT Transcription Complex, Subunit 1; also involved in the maintenance of embryonic cells). Since both genes are antisense with respect each other and have their 3' end overlapping, we can consider them as part of the same locus. Locus: the most relevant gene at that particular chromosomal region. Variant type: variant definition in GWAS Catalog. Protein: Protein names from Gene Cards webserver (24). Function: functional from GeneCards webserver. Function annotation provided by GeneCards.

Supplementary Figures

Supplementary Figure 1. Quantile - Quantile (QQ) plot of results from fixed-effects inverse variance-weighted-variance meta-analysis in 15,997 participants of Hispanic/Latino ancestry.

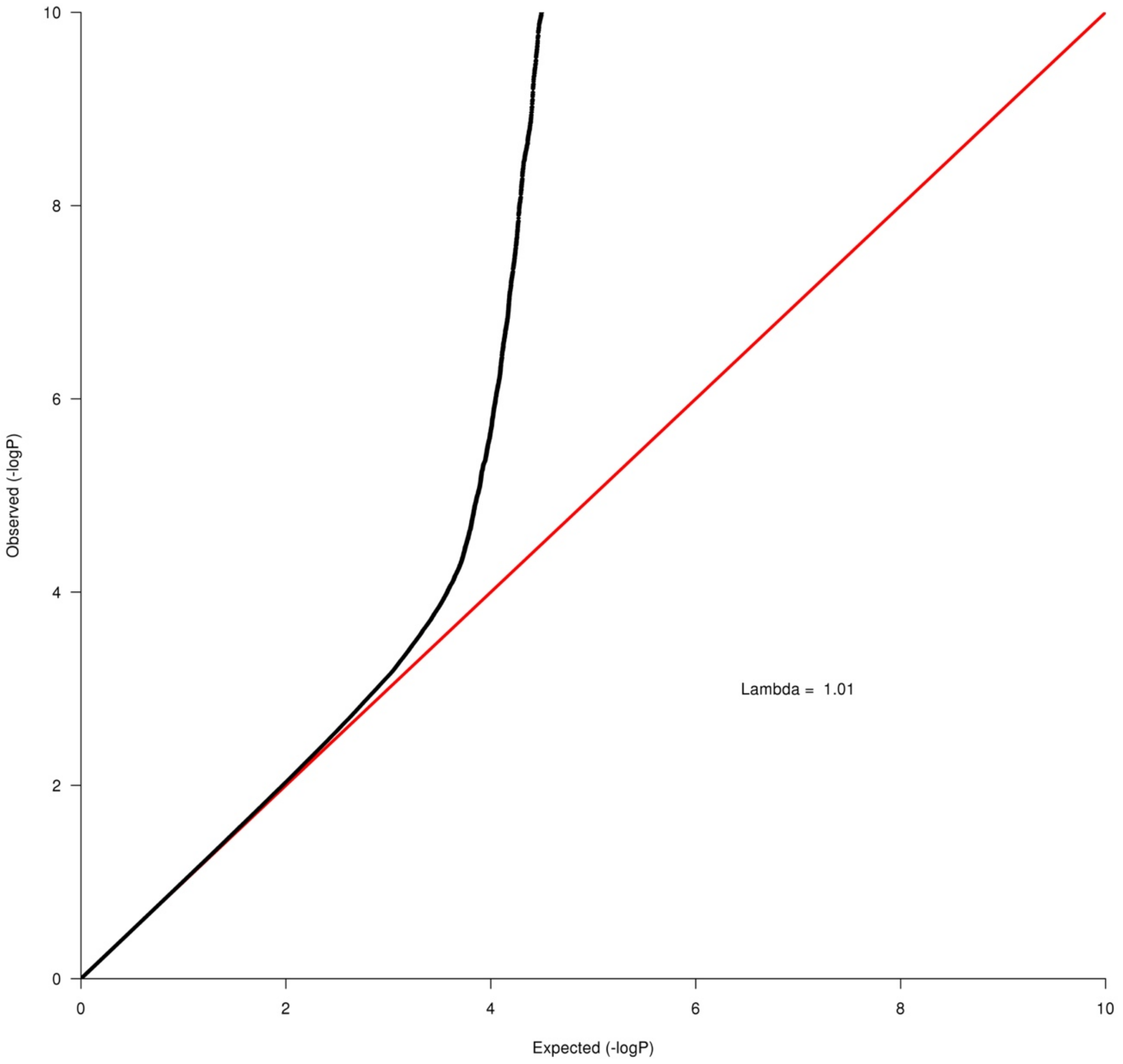

Supplementary Figure 2. QQ plots for the individual studies, prior to fixed effect, inverse-variant meta-analysis. Panel **A** is the QQ plot for the WHI study, panel **B** is the QQ plot for the MESA study, panel **C** is the QQ plot for the HCHS/SOL study and panel **D** is the QQ plot for the Starr County study.

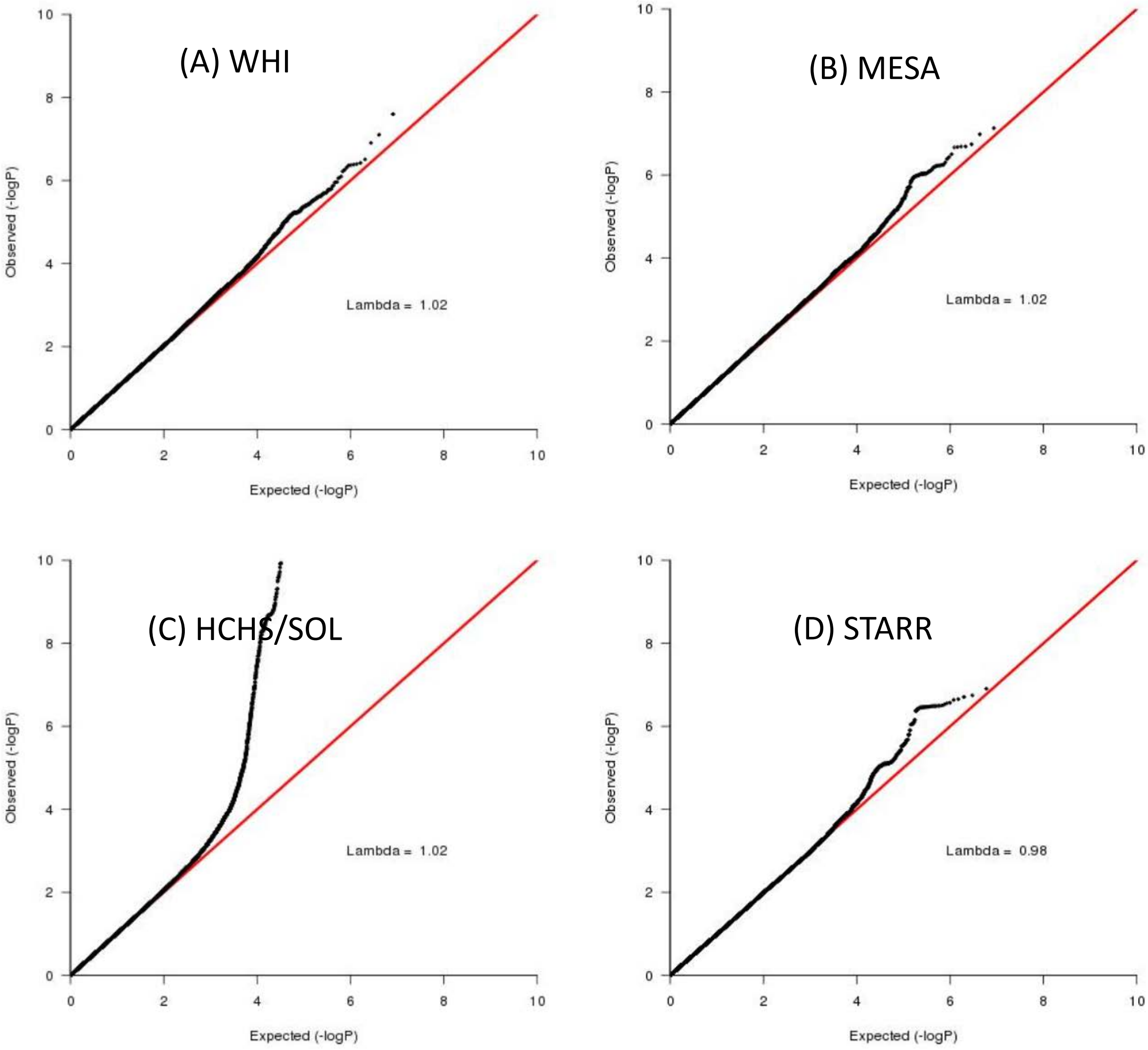

Supplementary Figure 3. Locus zoom plots for nine loci without evidence of secondary signals in 15,997 participants of Hispanic/Latino ancestry: *RNF207* (A), *SLC8A1* (B), *TTN* (C), *SLC35F1* (D), *KCNH2* (E), *LITAF* (F), *SETD6* (G), *PRKCA* (H), *KCNE1* (I).

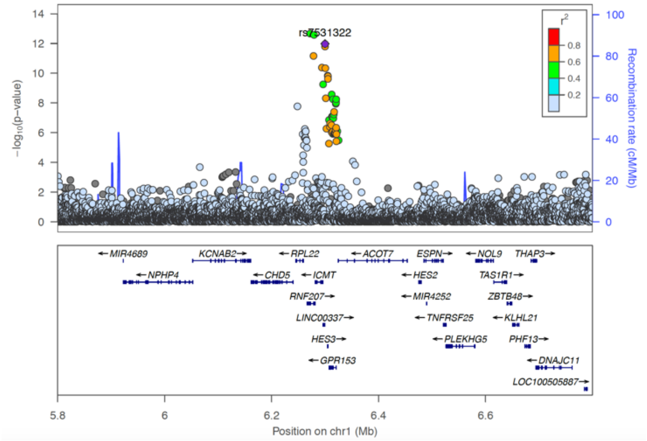

A

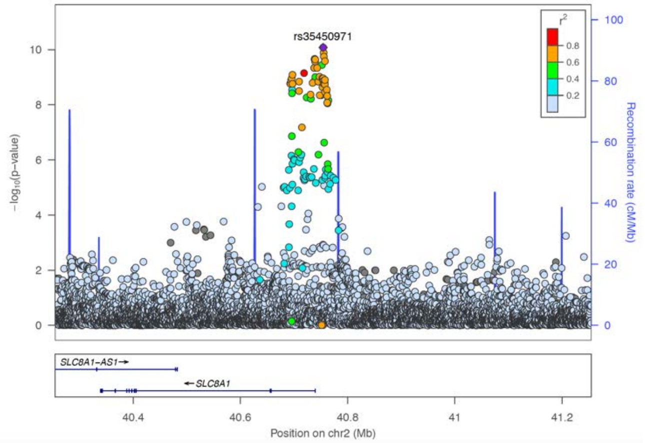

B

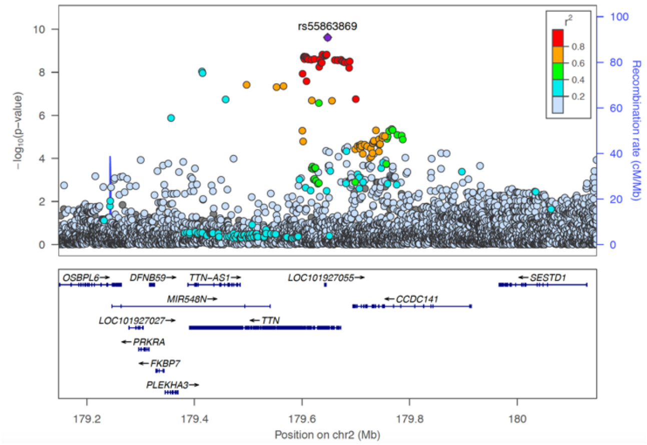

C

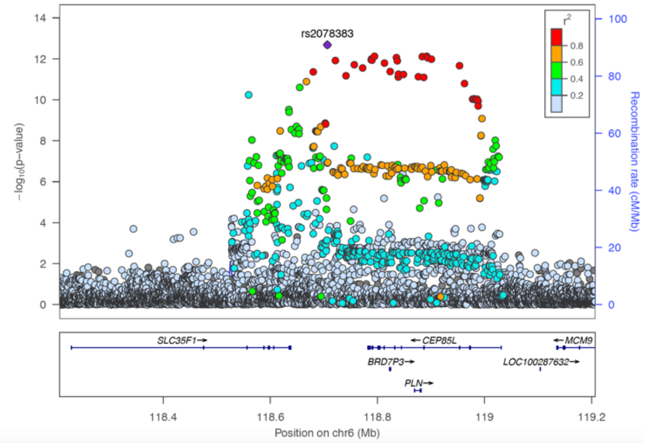

D

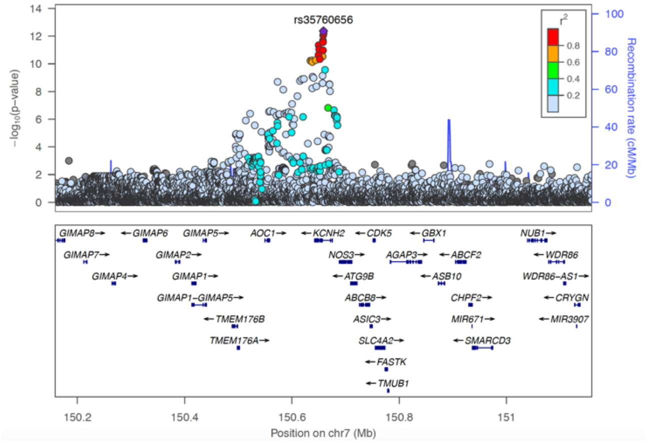

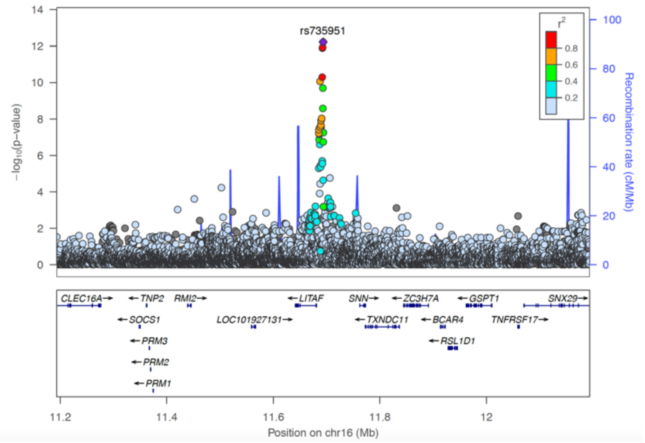

E

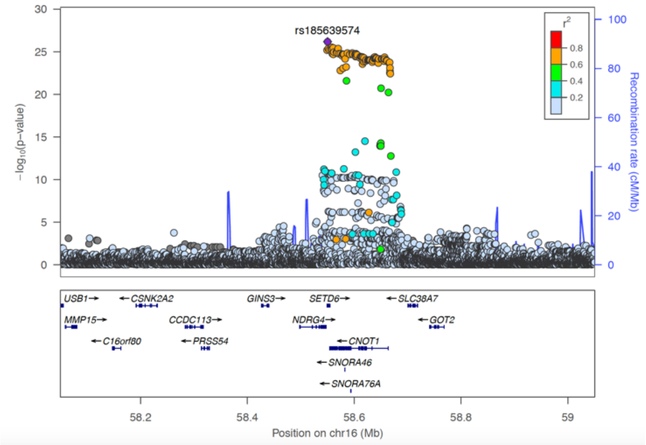

rs185639574
−log10(p−value)
Recombination rate (cM/Mb)
r2
0.8
0.6
0.4
0.2
USB1
CSNK2A2
GINS3
SETD6
SLC38A7
MMP15
CCDC113
NDRG4
GOT2
C16orf80
PRSS54
CNOT1
SNORA46
SNORA76A
Position on chr16 (Mb)

F

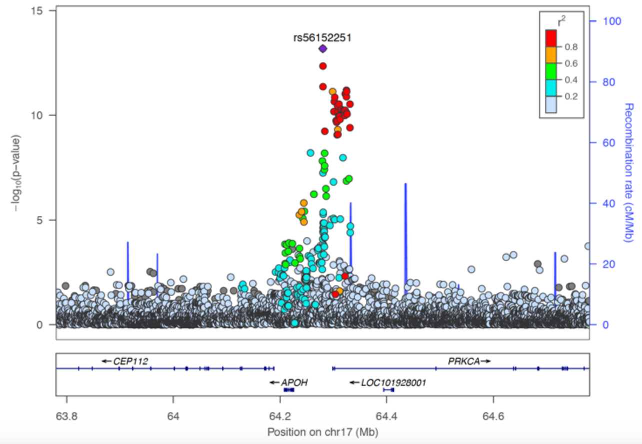

rs56152251
−log10(p−value)
Recombination rate (cM/Mb)
r2
0.8
0.6
0.4
0.2
CEP112
PRKCA
APOH
LOC101928001
Position on chr17 (Mb)

G

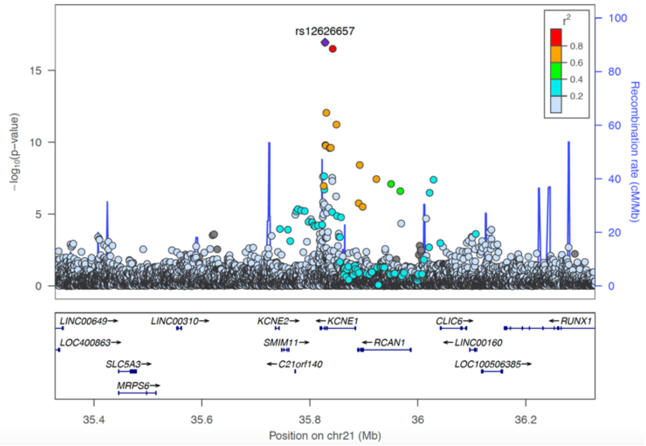

rs12626657
−log10(p−value)
Recombination rate (cM/Mb)
r2
0.8
0.6
0.4
0.2
LINC00649
LINC00310
KCNE2
KCNE1
CLIC6
RUNX1
LOC400863
SMIM11
RCAN1
LINC00160
SLC5A3
C21orf140
LOC100506385
MRPS6
Position on chr21 (Mb)

H

I

Supplementary Figure 4. Generalization of the 34 QT index SNPs identified in European populations to Hispanic/Latinos, controlling for the false discovery rate. Blue bars represent the confidence intervals for the beta estimates of index SNPs reported by Arking *et al.* (14) (dark blue) that generalized in Hispanics/Latinos (light blue). The red stars correspond to lead SNPs in Hispanics/Latinos that reached genome-wide significance. Red bars represent the confidence interval for the beta estimates of index SNPs that did not generalize in Hispanic/Latinos, with confidence intervals shown as orange bars.

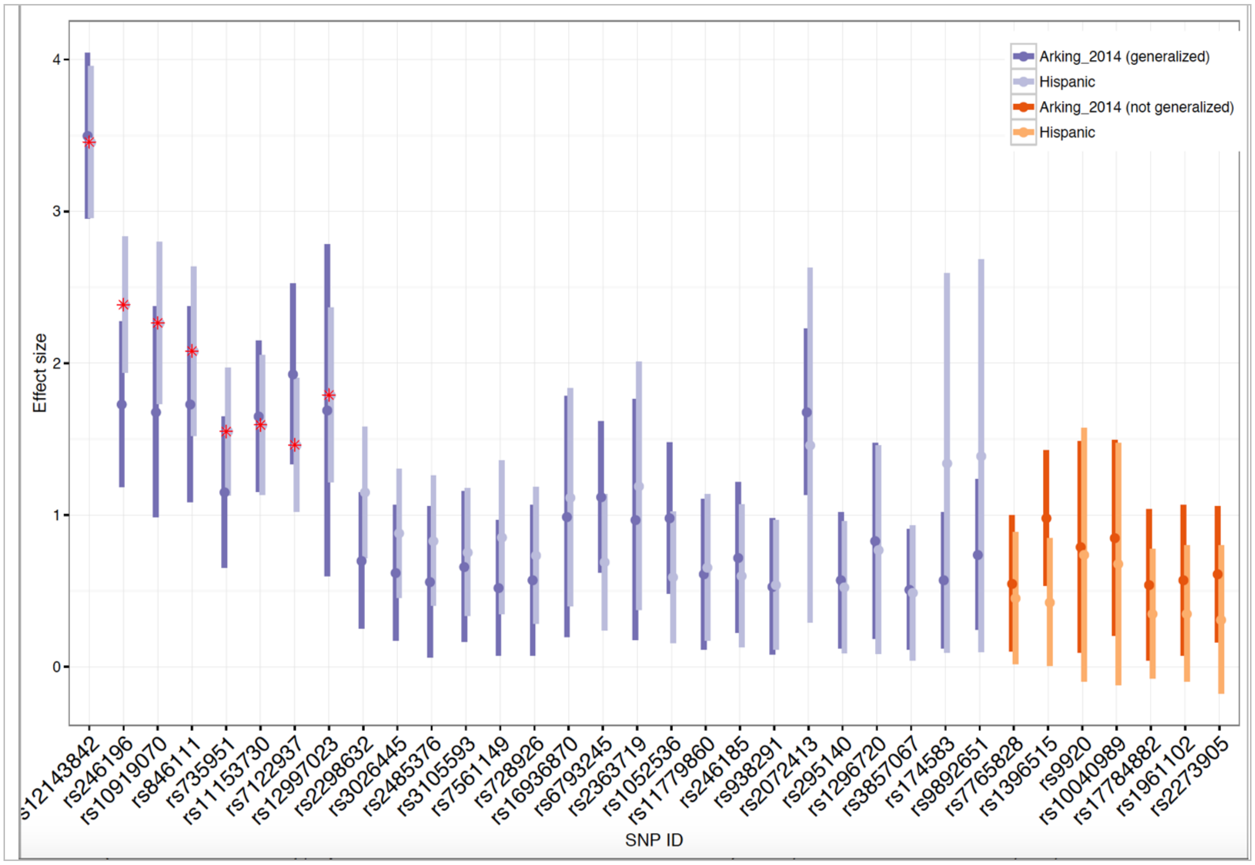

REFERENCES

1 Lavange, L.M., Kalsbeek, W.D., Sorlie, P.D., Aviles-Santa, L.M., Kaplan, R.C., Barnhart, J., Liu, K., Giachello, A., Lee, D.J., Ryan, J. *et al.* (2010) Sample design and cohort selection in the Hispanic Community Health Study/Study of Latinos. *Ann Epidemiol*, **20**, 642-649.

2 Sorlie, P.D., Aviles-Santa, L.M., Wassertheil-Smoller, S., Kaplan, R.C., Daviglus, M.L., Giachello, A.L., Schneiderman, N., Raij, L., Talavera, G., Allison, M. *et al.* (2010) Design and implementation of the Hispanic Community Health Study/Study of Latinos. *Ann Epidemiol*, **20**, 629-641.

3 Bild, D.E., Bluemke, D.A., Burke, G.L., Detrano, R., Diez Roux, A.V., Folsom, A.R., Greenland, P., Jacob, D.R., Jr., Kronmal, R., Liu, K. *et al.* (2002) Multi-ethnic study of atherosclerosis: objectives and design. *Am J Epidemiol*, **156**, 871-881.

4 Hanis, C.L., Redline, S., Cade, B.E., Bell, G.I., Cox, N.J., Below, J.E., Brown, E.L. and Aguilar, D. (2016) Beyond type 2 diabetes, obesity and hypertension: an axis including sleep apnea, left ventricular hypertrophy, endothelial dysfunction, and aortic stiffness among Mexican Americans in Starr County, Texas. *Cardiovasc Diabetol*, **15**, 86.

5 Below, J.E., Parra, E.J., Gamazon, E.R., Torres, J., Krithika, S., Candille, S., Lu, Y., Manichakul, A., Peralta-Romero, J., Duan, Q. *et al.* (2016) Meta-analysis of lipid-traits in Hispanics identifies novel loci, population-specific effects, and tissue-specific enrichment of eQTLs. *Sci Rep*, **6**, 19429.

6 WHI Study Group. (1998) Design of the Women's Health Initiative clinical trial and observational study. The Women's Health Initiative Study Group. *Control Clin Trials*, **19**, 61-109.

7 Staples, J., Nickerson, D.A. and Below, J.E. (2013) Utilizing graph theory to select the largest set of unrelated individuals for genetic analysis. *Genet Epidemiol*, **37**, 136-141.

8 Staples, J., Qiao, D., Cho, M.H., Silverman, E.K., University of Washington Center for Mendelian, G., Nickerson, D.A. and Below, J.E. (2014) PRIMUS: rapid reconstruction of pedigrees from genome-wide estimates of identity by descent. *Am J Hum Genet*, **95**, 553-564.

9 Welter, D., MacArthur, J., Morales, J., Burdett, T., Hall, P., Junkins, H., Klemm, A., Flicek, P., Manolio, T., Hindorff, L. *et al.* (2014) The NHGRI GWAS Catalog, a curated resource of SNP-trait associations. *Nucleic Acids Res*, **42**, D1001-1006.

10 Ward, L.D. and Kellis, M. (2016) HaploReg v4: systematic mining of putative causal variants, cell types, regulators and target genes for human complex traits and disease. *Nucleic Acids Res*, **44**, D877-881.

11 Ernst, J. and Kellis, M. (2012) ChromHMM: automating chromatin-state discovery and characterization. *Nat Methods*, **9**, 215-216.

12 Newton-Cheh, C., Eijgelsheim, M., Rice, K.M., de Bakker, P.I., Yin, X., Estrada, K., Bis, J.C., Marciante, K., Rivadeneira, F., Noseworthy, P.A. *et al.* (2009) Common variants at ten loci influence QT interval duration in the QTGEN Study. *Nat Genet*, **41**, 399-406.

13 Pfeufer, A., Sanna, S., Arking, D.E., Muller, M., Gateva, V., Fuchsberger, C., Ehret, G.B., Orru, M., Pattaro, C., Kottgen, A. *et al.* (2009) Common variants at ten loci modulate the QT interval duration in the QTSCD Study. *Nat Genet*, **41**, 407-414.

14 Arking, D.E., Pulit, S.L., Crotti, L., van der Harst, P., Munroe, P.B., Koopmann, T.T., Sotoodehnia, N., Rossin, E.J., Morley, M., Wang, X. *et al.* (2014) Genetic association study of QT interval highlights role for calcium signaling pathways in myocardial repolarization. *Nat Genet*, **46**, 826-836.
15 Marroni, F., Pfeufer, A., Aulchenko, Y.S., Franklin, C.S., Isaacs, A., Pichler, I., Wild, S.H., Oostra, B.A., Wright, A.F., Campbell, H. *et al.* (2009) A genome-wide association scan of RR and QT interval duration in 3 European genetically isolated populations: the EUROSPAN project. *Circulation. Cardiovascular genetics*, **2**, 322-328.
16 Nolte, I.M., Wallace, C., Newhouse, S.J., Waggott, D., Fu, J., Soranzo, N., Gwilliam, R., Deloukas, P., Savelieva, I., Zheng, D. *et al.* (2009) Common genetic variation near the phospholamban gene is associated with cardiac repolarisation: meta-analysis of three genome-wide association studies. *PLoS One*, **4**, e6138.
17 Holm, H., Gudbjartsson, D.F., Arnar, D.O., Thorleifsson, G., Thorgeirsson, G., Stefansdottir, H., Gudjonsson, S.A., Jonasdottir, A., Mathiesen, E.B., Njolstad, I. *et al.* (2010) Several common variants modulate heart rate, PR interval and QRS duration. *Nat Genet*, **42**, 117-122.
18 Kim, J.W., Hong, K.W., Go, M.J., Kim, S.S., Tabara, Y., Kita, Y., Tanigawa, T., Cho, Y.S., Han, B.G. and Oh, B. (2012) A common variant in SLC8A1 is associated with the duration of the electrocardiographic QT interval. *Am J Hum Genet*, **91**, 180-184.
19 Smith, J.G., Avery, C.L., Evans, D.S., Nalls, M.A., Meng, Y.A., Smith, E.N., Palmer, C., Tanaka, T., Mehra, R., Butler, A.M. *et al.* (2012) Impact of ancestry and common genetic variants on QT interval in African Americans. *Circ Cardiovasc Genet*, **5**, 647-655.
20 Sano, M., Kamitsuji, S., Kamatani, N., Hong, K.W., Han, B.G., Kim, Y., Kim, J.W., Aizawa, Y., Fukuda, K. and Japan Pharmacogenomics Data Science, C. (2014) Genome-wide association study of electrocardiographic parameters identifies a new association for PR interval and confirms previously reported associations. *Hum Mol Genet*, **23**, 6668-6676.
21 Chambers, J.C., Zhao, J., Terracciano, C.M., Bezzina, C.R., Zhang, W., Kaba, R., Navaratnarajah, M., Lotlikar, A., Sehmi, J.S., Kooner, M.K. *et al.* (2010) Genetic variation in SCN10A influences cardiac conduction. *Nat Genet*, **42**, 149-152.
22 Dalageorgou, C., Ge, D., Jamshidi, Y., Nolte, I.M., Riese, H., Savelieva, I., Carter, N.D., Spector, T.D. and Snieder, H. (2008) Heritability of QT interval: how much is explained by genes for resting heart rate? *J Cardiovasc Electrophysiol*, **19**, 386-391.
23 Newton-Cheh, C., Guo, C.Y., Wang, T.J., O'Donnell C, J., Levy, D. and Larson, M.G. (2007) Genome-wide association study of electrocardiographic and heart rate variability traits: the Framingham Heart Study. *BMC Med Genet*, **8 Suppl 1**, S7.
24 Fishilevich, S., Zimmerman, S., Kohn, A., Iny Stein, T., Olender, T., Kolker, E., Safran, M. and Lancet, D. (2016) Genic insights from integrated human proteomics in GeneCards. *Database (Oxford)*, **2016**.
